# An ancient tropical origin, dispersals via land bridges and Miocene diversification explain the subcosmopolitan disjunctions of the liverwort genus *Lejeunea*

**DOI:** 10.1038/s41598-020-71039-1

**Published:** 2020-08-24

**Authors:** Gaik Ee Lee, Fabien L. Condamine, Julia Bechteler, Oscar Alejandro Pérez-Escobar, Armin Scheben, Alfons Schäfer-Verwimp, Tamás Pócs, Jochen Heinrichs

**Affiliations:** 1 Faculty of Science and Marine Environment, University Malaysia Terengganu, 21030 Kuala Nerus, Terengganu Malaysia; 2 Institute of Tropical Biodiversity and Sustainable Development, University Malaysia Terengganu, 21030 Kuala Nerus, Terengganu Malaysia; 3 CNRS, UMR 5554 Institut des Sciences de l’Evolution de Montpellier, Place Eugène Bataillon, 34095 Montpellier, France; 4 Nees Institute for Biodiversity of Plants, University of Bonn, 53115 Bonn, Germany; 5 Comparative Plant and Fungal Biology, Royal Botanic Gardens, Kew, Richmond, TW9 3AB UK; 6 School of Biological Sciences, The University of Western Australia, Perth, WA 6009 Australia; 7 Unaffiliated, Herdwangen-Schönach, Germany; 8 Botany Department, Institute of Biology, Eszterházy University, Pf. 43, Eger, 3301 Hungary; 9 Department of Biology I, Systematic Botany and Mycology, Geobio-Center, University of Munich (LMU), Menzinger Str. 67, 80638 Munich, Germany

**Keywords:** Phylogenetics, Biogeography

## Abstract

Understanding the biogeographical and diversification processes explaining current diversity patterns of subcosmopolitan-distributed groups is challenging. We aimed at disentangling the historical biogeography of the subcosmopolitan liverwort genus *Lejeunea* with estimation of ancestral areas of origin and testing if sexual system and palaeotemperature variations can be factors of diversification. We assembled a dense taxon sampling for 120 species sampled throughout the geographical distribution of the genus. *Lejeunea* diverged from its sister group after the Paleocene-Eocene boundary (52.2 Ma, 95% credibility intervals 50.1–54.2 Ma), and the initial diversification of the crown group occurred in the early to middle Eocene (44.5 Ma, 95% credibility intervals 38.5–50.8 Ma). The DEC model indicated that (1) *Lejeunea* likely originated in an area composed of the Neotropics and the Nearctic, (2) dispersals through terrestrial land bridges in the late Oligocene and Miocene allowed *Lejeunea* to colonize the Old World, (3) the Boreotropical forest covering the northern regions until the late Eocene did not facilitate *Lejeunea* dispersals, and (4) a single long-distance dispersal event was inferred between the Neotropics and Africa. Biogeographical and diversification analyses show the Miocene was an important period when *Lejeunea* diversified globally. We found slight support for higher diversification rates of species with both male and female reproductive organs on the same individual (monoicy), and a moderate positive influence of palaeotemperatures on diversification. Our study shows that an ancient origin associated with a dispersal history facilitated by terrestrial land bridges and not long-distance dispersals are likely to explain the subcosmopolitan distribution of *Lejeunea*. By enhancing the diversification rates, monoicy likely favoured the colonisations of new areas, especially in the Miocene that was a key epoch shaping the worldwide distribution.

## Introduction

Intercontinental biogeographical disjunctions, in which species span several continents, have long been a main focus of evolutionary biologists and biogeographers. Since its inception, continental drift theory has provided exemplar systems in support of vicariance biogeography. However, the importance of vicariance in biogeography has been reconsidered since evidence for dispersal, and even long-distance dispersal (LDD), has accumulated from increasing numbers of molecular phylogenetic studies. Indeed, numerous studies have revealed that vicariance is not always the dominant factor explaining current distribution patterns in globally distributed organisms^1,2^. Consequently, historical biogeography now tends to systematically investigate the relative roles of dispersal and vicariance events to better understand global patterns of biodiversity distribution by the integration of molecular dating approaches.

Plants have been shown to be particularly good dispersers over geological timescales^2^. Among plants, bryophytes tend to have wider species distributional ranges than vascular plants^3^ and many bryophytes exhibit intercontinental distributions, which have been confirmed by molecular data^4–6^. The spores of bryophytes are considered to be very resistant to stress and can endure extreme temperatures, high levels of UV radiation, ultra-high pressure and long periods of desiccation^7,8^. Such adaptation has likely enabled the widespread distribution of many bryophyte species. Currently, phylogeny-based evidence rejects ancient vicariance events and favours LDD as the central driver shaping bryophyte global distributional patterns; such evidence includes the resilience of wind-dispersed spores^9,10^, the disjunct ranges of bryophytes as indicated by molecular phylogenies^11,12^ and, most recently, information from molecular dating and biogeographical analyses^13–15^. However, LDD alone does not always result in genetic divergence due to limitations in regard to strong gene flow, e.g., oceanic island diversity (anagenetic speciation^16^), and the strong geographical structure observed in species-level phylogenies is due to the high speciation rate within regions rather than LDD^17^.

Among bryophytes, the liverworts (Marchantiophyta) are monophyletic early-diverging land plants that may be the closest relatives of the two other bryophyte lineages (mosses and hornworts)^18–20^, possibly dating back to about 470 million years ago^21^ (Ma). Divergence time estimates of liverworts are greatly hindered by the scarcity of fossil records for this group^22^. Despite this limitation, time-calibrated molecular phylogenies have allowed the estimation of divergence times of major liverwort lineages. In the most species-rich family of liverworts, Lejeuneaceae, divergence-time estimation indicates the rapid establishment of the major lineages in the Cretaceous followed by steady diversification through the Cenozoic^23^. Although we have some knowledge regarding the early origin of Lejeuneaceae, we know little about their Cenozoic diversification, mostly because of a lack of well sampled, dated phylogenies at the species level. Studying the biogeography and diversification of Lejeuneaceae is difficult because the systematics and taxonomy remain poorly documented. The genus *Lejeunea* stands out as being one of the largest genera in Lejeuneaceae but the precise number of species remains unclear, with estimates from 200 to 300 species^24,25^. The uncertainties arise from the unstable circumscription of the genus limit, which has considerably expanded (the inclusion of 25 genera in *Lejeunea*^26^), and the approximately 200 unrevised names of *Taxilejeunea* of Neotropical origin, a genus now considered as a synonym of *Lejeunea*. Because of their relatively well-supported relationships, comprehensive taxon sampling and broad geographical distribution, the genus represents an ideal candidate for exploring several hypotheses explaining subcosmopolitan distribution and general patterns of Cenozoic diversification. Previous studies estimated that the genus diverged in the early Cenozoic and that the early-diverging lineages occurred in the Neotropics^27^. However, we still know very little about the evolutionary history of the genus, and the diversification processes over space and time remain unknown.

Here, we study the historical biogeography and diversification of the genus *Lejeunea*. We first ask whether the current *Lejeunea* distribution can be explained with vicariance or dispersal events, or a combination of both. Given their high dispersal ability (resistant spores), we predict that continentally restricted clades are relatively young and did not originate by vicariance but instead through dispersal and possibly LDD. Alternatively, the ancient origin of *Lejeunea*^22,23^ and apparent early divergence of the main lineages in the New World^27,28^ may suggest that the genus reached a global distribution through terrestrial land bridges such as the Bering land bridges (BLB) or the North Atlantic land bridges (NALB) with the Thulean and De Geer routes, which contributed to the expansion of the Boreotropical forest in the Eocene^29–31^. We carried out molecular dating and biogeographical analyses to estimate the divergence times and ancestral ranges of *Lejeunea*. Second, we investigated possible drivers of diversification by asking: Do the sexual systems of *Lejeunea* (monoicy and dioicy) and/or past temperatures correlate with the diversification rates? We expect that monoicous species have higher diversification rates than dioicous species^32^, and that past temperatures have influenced species diversification because palaeoclimate change is considered as a main trigger of diversification among many terrestrial organisms^33,34^. Palaeotemperature and tolerance to cold can also be considered as the main factors conditioning dispersal success over the Beringian and Transatlantic land bridges^1^. We used trait-dependent and environmental-dependent models to assess the correlations of those factors with the diversification of *Lejeunea*.

## Results

### Phylogenetic relationships and divergence times

The maximum likelihood (ML) analyses and the Bayesian inference (BI) of phylogeny indicated *Lejeunea* as a well-supported clade with a bootstrap value (BV) of 80% and a posterior probability (PP) of 1.0 (Supplementary Fig. S1). The six representatives of *Harpalejeunea* formed a monophyletic group as well as the 16 representatives of *Microlejeunea,* and the latter genus was placed as sister to *Lejeunea*, comprising 120 species. The monophyly of *Harpalejeunea*, *Microlejeunea* and *Lejeunea* and the sister relationship of the two latter genera were similar to those obtained by Heinrichs et al*.*^27^. The *Lejeunea* clade was divided into two well-supported main lineages, and the subgenus assignment was based on Heinrichs et al*.*^27^, corresponding to *Lejeunea* subg. *Lejeunea* (BV = 100%; PP = 1.0) and *Lejeunea* subg. *Crossotolejeunea* (BV = 75%; PP = 0.99). We further identified six major clades in the crown group (see Supplementary Fig. S1 for BV and PP values).

**Figure 1:**
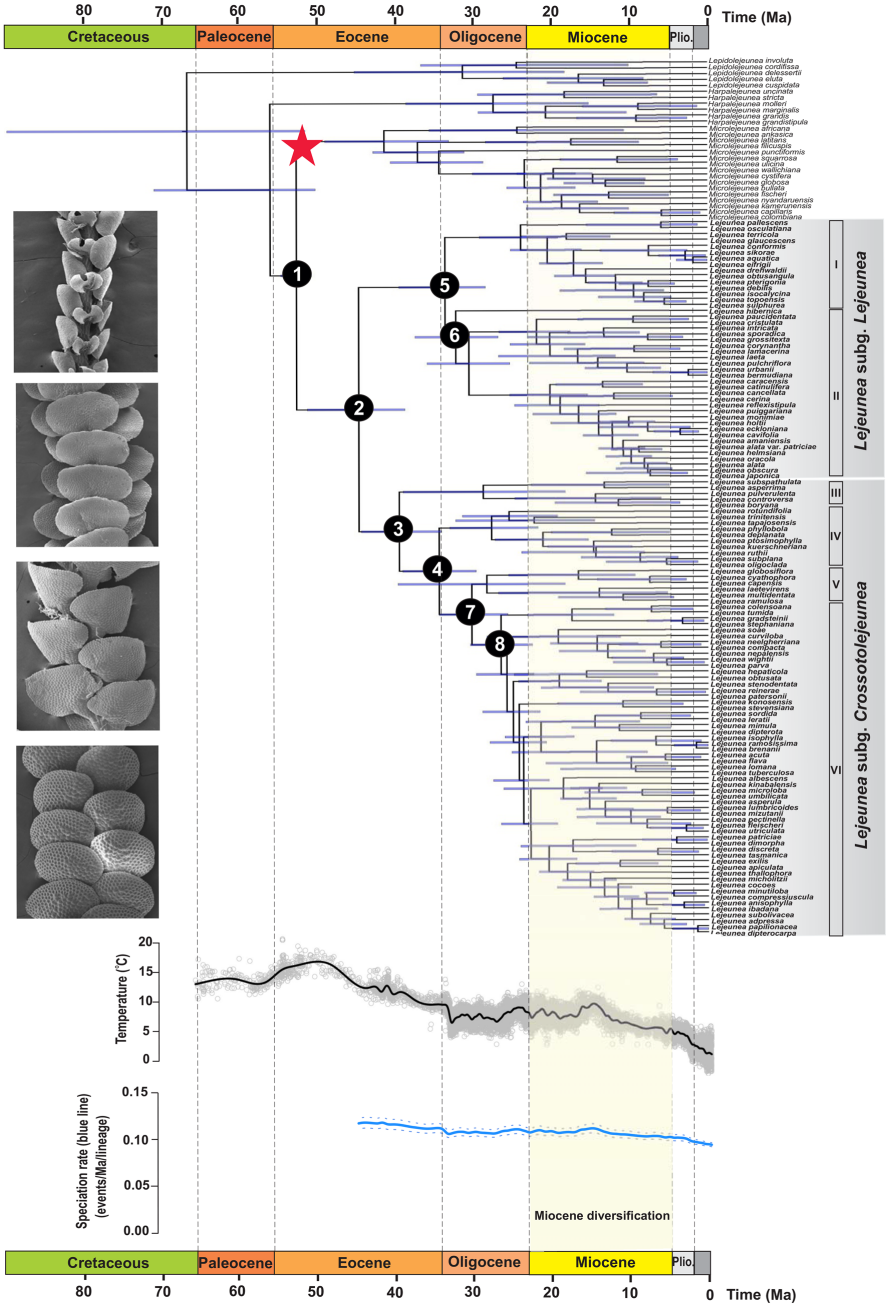
Time-calibrated tree and global diversification pattern of *Lejeunea*. The chronogram tree indicates mean ages and node bars show the 95% credibility intervals. Red star indicates fossil calibration. Scanning electron micrographs show representatives of *Lejeunea*; from top: *Lejeunea stephaniana*, *L. mimula* in ventral view, and *L. kinabalensis*, *L. umbilicata* in dorsal view. Paleoenvironment-dependent diversification processes in *Lejeunea*, indicating a weak positive correlation between temperatures and speciation rates. Nodes 1–8 are summarized in Table 1. (Plio., Pliocene).

The divergence-time estimates obtained under a strict-clock and a relaxed-clock model produced similar results with mean age estimates differing by less than 1.6 million years at the eight deepest nodes between the two analyses (Table 1). The phylogenetic tree of *Lejeunea* generated with BEAST (Fig. 1) was consistent with the topologies from the ML and Bayesian analyses except the nodes with low BV and PP values. The uncorrelated relaxed-clock model suggested an origin of the *Lejeunea* stem lineage in the early Eocene (52.2 Ma; 95% credibility intervals [CI] 50.1–54.2 Ma; node 1 in Fig. 1) and the earliest diversification of the *Lejeunea* crown group in the middle Eocene (44.5 Ma; 95% CI 38.5–50.8 Ma; node 2 in Fig. 1), followed by the subgenus *Crossotolejeunea* crown group in the late Eocene (39.2 Ma; 95% CI 33.8–44.0 Ma; node 3 in Fig. 1). The diversification of other major clades occurring in the Oligocene and Miocene and the ages of the clades within *Lejeunea* identified in our study are summarized in Table 1. Divergence times estimation with a normal distribution prior and a standard deviation of 5 Ma is presented in Supplementary Table S1 in which the *Lejeunea* stem was inferred at 54.9 Ma (95% CI 46.3–63.8 Ma).

**Table 1:** Divergence time estimates of main clades of *Lejeunea* obtained from four analyses in BEAST.

Node	RC, birthdeath	RC, Yule	SC, birthdeath	SC, Yule	Substitution rate
1. *Lejeunea* stem	52.2 (50.1–54.2)	52.1 (50.1–54.0)	52.1 (50.1–54.0)	52.0 (50.0–53.9)	63.3 (41.6–87.4)
2. *Lejeunea* crown	44.5 (38.5–50.8)	43.4 (35.6–52.2)	43.5 (35.6–52.0)	42.2 (36.0–51.1)	52.0 (35.1–71.4)
3. *Lejeunea* clade III crown	39.2 (33.8–44.0)	37.0 (31.3–42.9)	37.2 (31.1–42.7)	35.8 (31.7–40.0)	46.3 (30.9–63.7)
4. *Lejeunea* clade IV crown	34.1 (29.4–38.8)	31.7 (26.8–36.7)	31.8 (27.0–36.9)	31.3 (28.0–34.8)	40.0 (27.0–54.8)
5. *Lejeunea* clade I crown	33.4 (28.2–39.2)	31.2 (25.2–37.4)	31.2 (25.7–37.4)	29.1 (25.0–33.0)	39.2 (26.0–51.1)
6. *Lejeunea* clade II crown	32.0 (26.6–37.1)	30.0 (24.7–35.2)	30.0 (24.4–35.4)	27.0 (23.5–30.6)	37.4 (24.0–51.1
7. *Lejeunea* clade V crown	30.0 (35.4–34.1)	27.5 (23.0–32.0)	27.6 (23.2–32.2)	26.6 (23.4–30.0)	35.6 (23.4–49.2)
8. Lejeunea clade VI crown	26.3 (22.3–30.2)	26.5 (22.4–31.0)	26.5 (22.4–30.1)	23.2 (20.5–26.1)	30.6 (20.6–41.9)
Mean posterior value	− 36,049.0	− 36,274.2	− 36,273.2	− 36,466.9	− 36,080.7
Log marginal likelihood	− 36,492.2	− 36,508.0	− 36,510.8	− 36,583.9	− 36,490.9

*RC, Birth–Death* Relaxed clock with Birth–Death tree including incomplete sampling,*RC, Yule* Relaxed clock using Yule parameter, *SC, Birth–Death* Strict clock with Birth–Death tree including incomplete sampling, *SC, Yule* Strict clock using Yule parameter.

### Biogeography and diversification of *Lejeunea*

**Figure 2:**
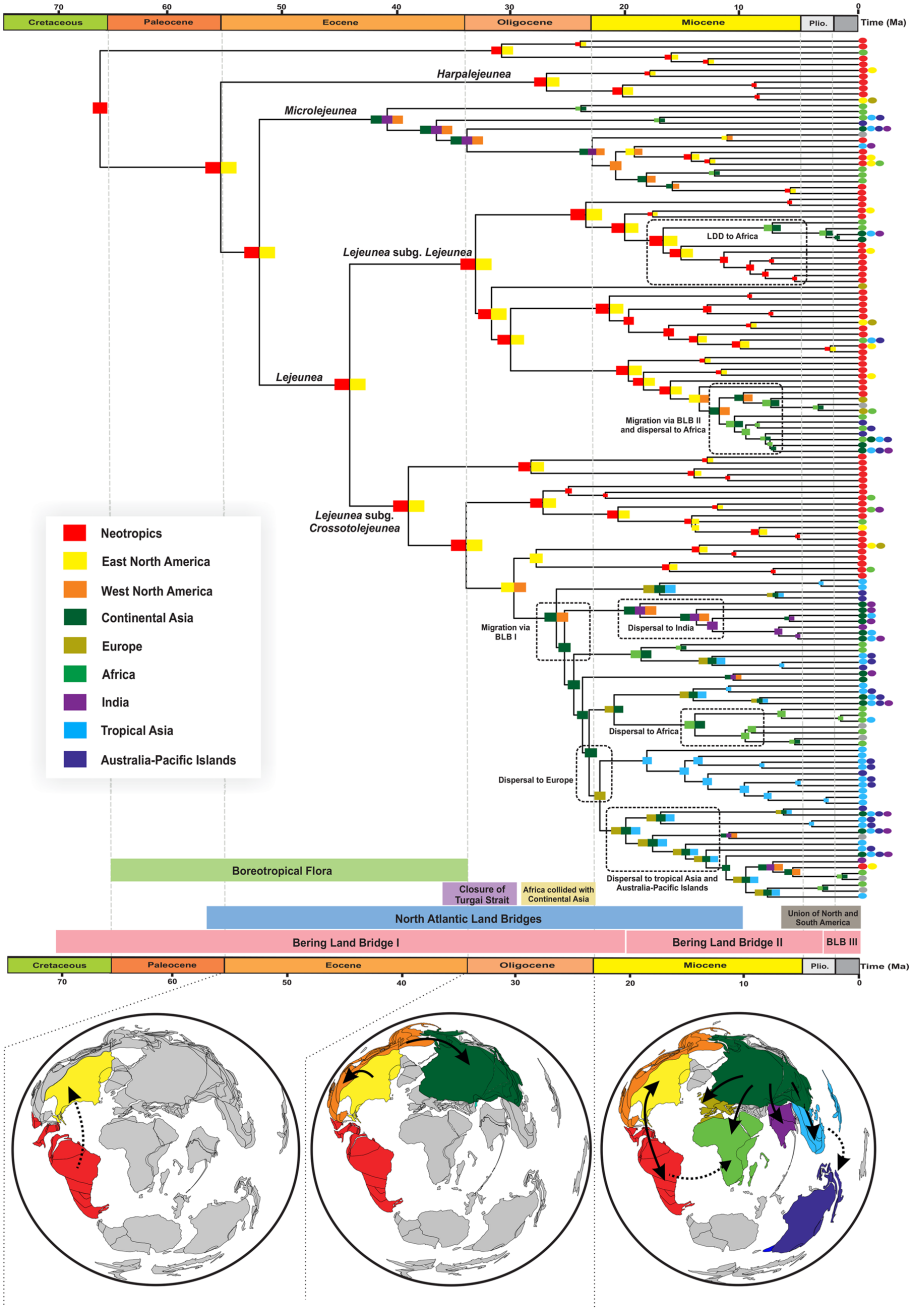
Reconstruction of historical biogeography of *Lejeunea* using a time-stratified DEC model as inferred from BioGeoBEARS. The below-left box showing nine biogeographical regions in colours as defined in this study. For each node, a coloured square represents the inferred area/areas with the highest relative probability in the DEC analysis while the coloured circles at the tips indicate the present day distributions of each species. Dashed boxes represent biogeographical patterns that are explained in the text. (Plio., Pliocene).

The results of both models of ancestral-area estimations (unconstrained and time-stratified) are presented in Supplementary Table S2. Estimations under the unconstrained DEC model (Supplementary Fig. S2) inferred the Neotropics as the ancestral area of *Lejeunea* and revealed at least seven lineages that have reached Europe, Africa and Tropical Asia, six lineages from Tropical Asia to Africa and Australia-Pacific Islands, and at least two lineages dispersed from Africa to Australia-Pacific Islands. Most of the disjunction between the Paleotropics and the Neotropics occurred later in the Miocene. The results of the time-stratified analyses (Fig. 2, Supplementary Fig. S3) indicating an ancestral area including the Neotropics and eastern North America. Two intercontinental dispersal events between western North America and continental Asia were inferred to have occurred during the Oligocene and Miocene. The ancestral-area estimation also showed six dispersal events from continental Asia to Europe, Africa, tropical Asia and the Australia-Pacific Islands in the Miocene to Pliocene. Dispersal to Europe occurred most recently around the early Miocene. The dispersal rate of *Lejeunea* of 0.0053 and 0.1483 per Ma and extinction rate of 0.0002 and 0.0583 were estimated under the unconstrained model and the time-stratified model, respectively.

The palaeoenvironment-dependent analyses indicated that the best-fit model includes speciation rates of *Lejeunea* that were positively, albeit weakly (α = 0.0013), associated with temperature fluctuations over the past 50 million years (Supplementary Table S3). This result suggests that speciation was higher during periods of global warming (Fig. 1). However, the difference of fit between the palaeoenvironment-dependent model and the constant-rate model is not significant (∆AIC = 0.669), and the standard deviations (± 1.126) is almost twice as large as the difference between the two models (Supplementary Table S3).

**Figure 3:**
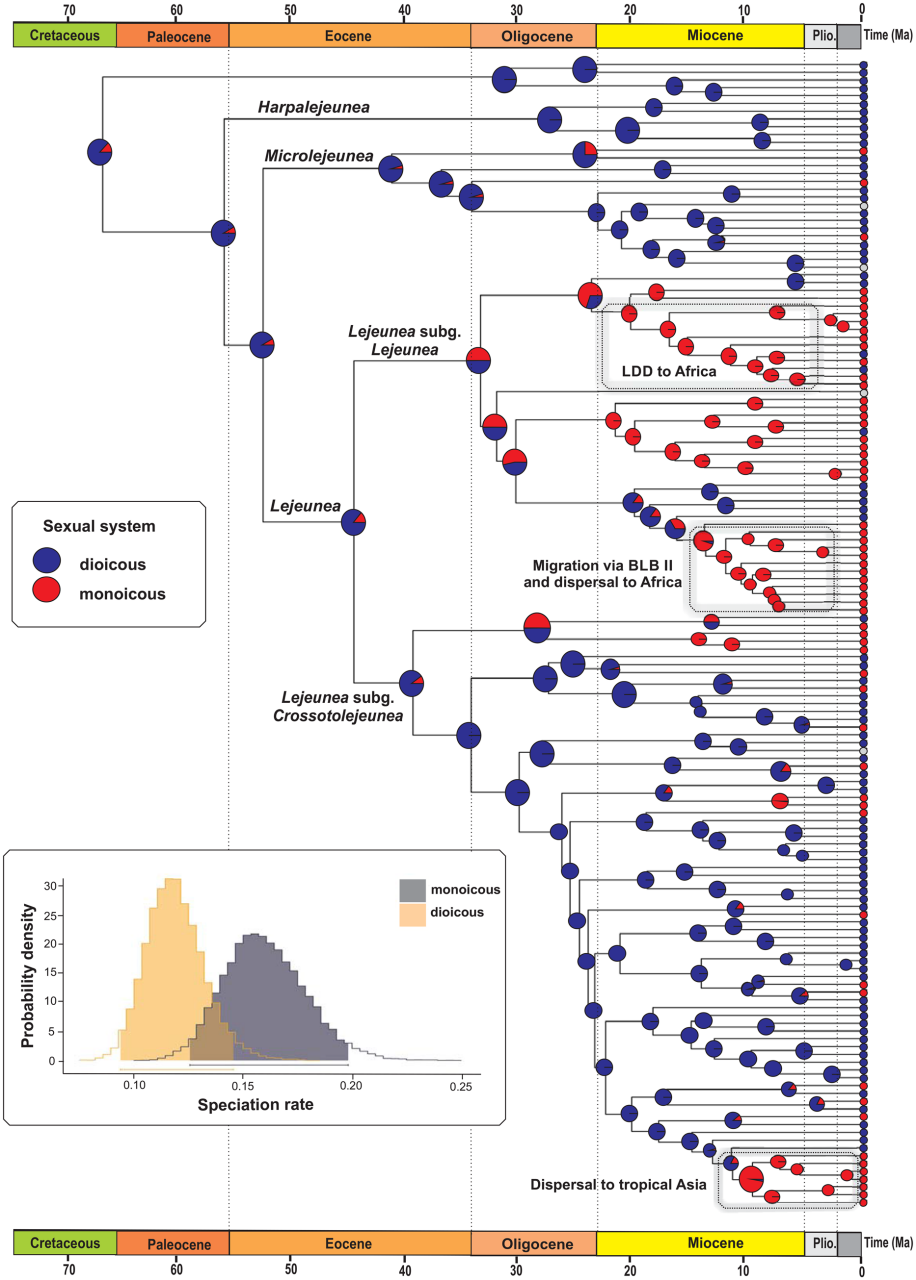
Ancestral character estimation of sexual system of *Lejeunea* with the proportional likelihoods mapped in pie diagrams above nodes. States for each terminal node are given according to the following traits of sexual system (dioicous: blue) and (monoicous: red). Unknown character states are in grey. Posterior probability distributions of the speciation rates from the BiSSE analysis showing the speciation rates were higher in monoicous lineages. Dashed boxes represent dispersal/migration of *Lejeunea* lineages that are explained in the text. (Plio., Pliocene).

The trait-dependent analyses with the BiSSE model revealed that the speciation rates were higher in monoicous species than in dioicous species, while the extinction and transition rates were equal (Fig. 3, Supplementary Table S4). The model estimated a net diversification rate of 0.1298 events per million years per monoicous lineage and of 0.0941 for dioicous lineages. Randomization analyses with BiSSE show that the null distribution of BiSSE ΔAIC values obtained from analyses with reshuffled states is centred towards values of 5 (Supplementary Fig. S4), which is close to the ΔAIC values obtained under analyses with real states, suggesting that we cannot exclude type-I error in these analyses. HiSSE analyses indicated that the CID-2 model is not supported against the best BiSSE model (mean LogL =  − 516.947 and mean AICc = 1,044.417 computed over 500 posterior trees for the CID-2 model, ∆AIC = 13.95).

## Discussion

Our data and biogeographical analyses suggested that the early-diverging lineages of *Lejeunea* were present in either the Neotropics or eastern North America in the middle Eocene. We hypothesize that the ancestral origin of *Lejeunea* is most likely to be in the Neotropics because (1) the highest extant species richness occurs in the Neotropics^35^, (2) the early-diverging lineages are found in the Neotropics (Fig. 2), and (3) all *Lejeunea* fossils have only been found in Miocene amber from the Dominican Republic^36,37^, although Paleogene ambers from Europe contain Lejeuneaceae fossils that are not attributed to *Lejeunea* so far^22^. The hypothesis of a Neotropical origin is also consistent with the ancestral-area estimation by Heinrichs et al*.*^27^ and a biogeographical model without palaeogeographical constraints. However, the “out of North America” hypothesis cannot be ruled out merely due to the lower species richness, as the region experienced severe extinction events with global cooling in the late Eocene and Oligocene and contains recognized Boreotropical elements^1^. The hypothesis regarding the ancestral area of *Lejeunea* could be further tested using more complete sampling of taxa because the current dataset includes only 40–60% of the extant species diversity (approximately 200–300 species).

Our dating analyses estimated divergence times of 26.3 Ma (95% CI 22.3–30.2 Ma) and 12.26 Ma (95% CI 8.83–15.8 Ma) for the two dispersals between North America and continental Asia. The late Oligocene and middle Miocene correspond well with the onset of the Bering Land Bridges^31^ (BLB) and the North Atlantic land bridges^38^ (NALB). Geological and paleobotanical evidence suggest a land connection between North America and Asia through the BLB in the Oligocene with 82% of plant genera distributed across the land bridge^39^. Recently, Jiang et al.^40^ revealed a long-lasting phase of biotic interchange, with a peak of dispersal from Asia into North America during the late Oligocene warming (26–24 Ma). Although the genus *Lejeunea* is thought to be prone to LDD (Supplementary Table S2, Fig. S2), we inferred terrestrial dispersal through existing land bridges for explaining the early geographical distribution of *Lejeunea.* Given that the divergence time estimate is congruent with the geological records of appropriate land connections and the tropical habitat during Oligocene and Miocene, we favour the hypothesis of terrestrial dispersal via land-bridges route rather than LDD. The clade crown group of *Lejeunea* appears to have been dispersed across the Northern Hemisphere at least twice via the BLB in the Oligocene and Miocene while such route was not employed by *Lejeunea* stem and crown group in the Eocene, which coincided with the Boreotropical flora^1,29,30^. Many elements of the Boreotropical flora became extinct across the Northern Hemisphere following the global climatic deterioration beginning in the Oligocene onward^41^. Such a geographical extirpation of biodiversity offered evolutionary opportunities in which many vascular plant groups underwent remarkable radiation and/or rapid diversification at lower latitudes, such as several angiosperm lineages (Annonaceae^42^, Malpighiaceae^43^, and Meliaceae^44^) and ferns (*Asplenium*^45^*, Diplazium*^46^ and *Nephrolepis*^47^). However, Boreotropical migration and extinction remain poorly known in liverworts. This is partly due to the absence of high-latitude fossil records or Boreotropical fossils. The low fossilization rate of bryophytes or the old age of Palaeotropical amber deposits containing liverworts fossils^22^ could be the main reasons for the absence of Boreotropical fossils in liverworts. The dispersal events reconstructed at the *Lejeunea* crown group (node 2 in Fig. 1) is timed at 38.5–50.8 Ma, matching the presence of the Boreotropical flora from the Palaeocene to the Eocene^29,30^. However, the Eocene Indian amber fossil does not indicate an ancient occurrence of early-divergent lineages of *Lejeunea* in high latitudes of the Northern Hemisphere, which does not support an early Boreotropical migration route. Instead, our ancestral-area estimation shows a post Boreotropical dispersal through northern land bridges (more likely the BLB) in the Oligocene and Miocene. Contrary to *Lejeunea*, the early diversification of *Microlejeunea* (sister to *Lejeunea*) occurred in the Northern Hemiphere around 40.8 Ma (95% CI 38.5–50.8 Ma) with the migration from North America to Continental Asia that likely went through the BLB (Fig. 2), which supports *Microlejeunea* as a member of the Boreotropical flora.

The Boreotropical migration and land bridges such as BLB and NALB played very important roles in the floristic and faunal interchanges within the Northern Hemisphere, particularly between eastern Asia and Western North America during the Eocene to Miocene^38,39^. Many vascular plant migrations across the land bridges were supported by both the fossil record and phylogenetic analyses addressing the Late Cretaceous to late Neogene^39,40^ (see review in Wen et al.^48^). In contrast, nonvascular plants, such as bryophytes, have received little attention regarding their migration across the land bridges, and no phylogenetic study has analysed such distribution patterns with biogeographical tools except Beringia considered as a refugium for *Sphagnum orientale*^49^.

In the present study, the diversification of *Lejeunea* was postulated in continental Asia and later occurred in Europe, tropical Asia, India and Africa in the early to late Miocene, and all the remaining disjunct distributions were located in continental Asia or across several continents. The migration and/or dispersal events finally led to the distribution and colonization of *Lejeunea* globally. The highest number of migration events, as expected, was found between the Neotropics and eastern North America, followed by migration from continental Asia to Africa. The possibility of biotic exchange in the latter migratory route is due to the union of the African-Arabian plate with continental Asia, which interrupted the Tethys Sea in the Miocene and therefore enabled plant and animal exchange.

Alternative migration routes from continental Asia to Europe might have been available in the middle Miocene, when the Turgai Sea closed up approximately 30 Ma^50^; the presence of this sea is considered as a barrier biotic exchange between Europe and continental Asia. The oldest colonization of Europe from Asia dates back to the Oligocene–Miocene boundary (Fig. 2), which postdates the closure of the Turgai Sea. Colonization of tropical Asia and the Australia-Pacific Islands occurred more recently during the Pliocene and Miocene. The dispersal towards tropical Asia could be the result of LDD or land connection dispersal across the Arabian corridor, where relatively mesic conditions prevailed. The presence of dispersal agents, such as migratory birds^51^, mammals (roe deer or wild boar^52^), and wind^9^, could provide evidence in favour of the former hypothesis.

Our results also show that all the early-diverging lineages, including those that arose via crown diversification, occurred in the early to middle Oligocene, while the remaining disjunct distribution events were more recent and mostly restricted to the Miocene and Pliocene. The origin and early divergence of *Lejeunea* occurred before the maximum temperature peaked in the Eocene, while major assemblies arose during the large-scale global cooling and aridification in the Miocene period. The establishment of the topography, climate and geography of the modern world took place in the Miocene along with numerous dramatic changes, including periods of volcanism, the uplift and formation of mountains, and the expansion of tropical forests^53^, which in turn promoted species diversification (see Nie et al*.*^54^ and references therein). The Miocene was a period that underwent the renewed expansion of megathermal forests^55^, which could explain the observed pattern in the distribution of *Lejeunea*. *Lejeunea* species show broad ecological ranges and are mostly epiphytes or even grow epiphyllously on vascular plant leaves. During the Miocene, vascular plants were already established and could have created suitable microhabitats in which *Lejeunea* species could have diversified.

In this study, it was initially hypothesized that species diversification was positively associated with past temperatures. Our palaeotemperature-dependent analysis shows that past temperatures did slightly affect the speciation rates of *Lejeunea* such that global warming fostered species diversification. Although global temperatures have likely enhanced the diversification of numerous plant families^56,57^, they had surprisingly weak effect on the diversification of *Lejeunea* (Fig. 1). It is important to be cautious about this result because the difference of model support between the best-fit model and the second best-fit model is weak. Although this is the first well-sampled dated phylogeny of *Lejeunea*, we remind that the taxon sampling remains overall low to infer a robust diversification history. Nonetheless, the best temperature-dependent model translates into higher speciation during the emergence of the East Asian summer monsoons in the Miocene *ca*. 23 Ma^57^, which provided a relatively warm and wet climate with high precipitation (see review in Tada et al.^58^), and also during the mid-Miocene climatic optimum (15–17 Ma) event. Two features of bryophytes may explain the weak effect of temperature-dependence. First, *Lejeunea*, and bryophytes in general, are very small in size, and their distributions are mainly dependent on the microenvironment rather than on macroclimatic characteristics^59^. Second, unlike vascular plants, bryophytes are poikilohydric and they are able to tolerate dehydration and to recover from it without physiological damage. This is a successful life strategy that allows resisting desiccation and surviving under cold and dry conditions. Although palaeoclimate change has been proven to be the main trigger of diversification among many terrestrial organisms^33,34^, in our study the past global temperatures seem unlikely to have played a dominant role in the diversification of *Lejeunea*. In future macroevolutionary studies of *Lejeunea*, this hypothesis can be tested again with novel data and models. Furthermore, the Cenozoic climate change likely impacted the biogeographical pattern of *Lejeunea*. After dispersing through the BLB, we found that several dispersal events were synchronized with global warming: dispersals to Europe and India during the late Oligocene warming event, and dispersals to Africa, tropical Asia, and Australia during the mid-Miocene climatic optimum (Fig. 2).

The BiSSE analyses show that the monoicous state spurred the diversification of *Lejeunea* lineages (Fig. 3), while the turnover was higher for dioicous species. Higher diversification rates in bisexual lineages have been previously reported in several studies, for instance in liverworts^32^ and mosses^60^. Several authors have suggested a correlation between the sexual system of bryophytes and their geographical range, in which monoicous species may have wider ranges than dioicous species based on spore production^61^. However, instead of the expected significant correlation between sexual system and range size, Laenen et al*.*^62^ demonstrated the importance of vegetative propagules in enhancing the LDD of bryophytes. The sexual systems of *Lejeunea* (51% dioicous, 48% monoicous in the present study) are more phylogenetically dependent, and the two clades demonstrated a rather stable and fixed bisexual and unisexual situation. An uneven distribution was similarly found in the clade *Lejeunea* subgenus *Lejeunea*, in which most of the species are monoicous. In contrast, in the *Lejeunea* subgenus *Crossotolejeunea*, dioicous species are predominant. Both the monoicous and dioicous species are capable of forming disjunct ranges; however, monoicy likely facilitated the dispersal/migration of *Lejeunea* lineages, e.g., LDD to Africa and migration via BLB II in the *Lejeunea* subgenus *Lejeunea* clade and at least one dispersal event to tropical Asia in the *Lejeunea* subgenus *Crossotolejeunea* clade (Fig. 3). Similarly to temperature models, our hypothesis can be tested and challenged in future studies focusing on the role of sexual system on the diversification of *Lejeunea*.

## Conclusion

Studying the processes shaping clades’ global distribution remains difficult, requiring a combination of multiple approaches. In summary, the evolutionary history of *Lejeunea* shows several biogeographical scenarios in terms of shaping the global distribution of the genus. Abiotic and biotic factors in relation to diversification also play important roles in the expansion of the genus. The unusual northern routes of colonization through the BLB and NALB may have facilitated the dispersal from North America to continental Asia at least twice during the Oligocene and Miocene. A global colonization pattern during the Miocene-Pliocene seems to be a common feature observed in many groups of organisms that exhibit worldwide distribution such as the subcosmopolitan distribution of *Lejeunea*.

## Materials and methods

### Taxon sampling and DNA extraction, amplification, sequencing and alignment

We sampled over the entire geographical distribution of the genus to obtain species from all continents. In total the dataset included 120 species of *Lejeunea*. Total genomic DNA was isolated using the Invisorb Spin Plant Mini Kit (Stratec Molecular GmbH, Berlin, Germany) prior to amplification. The *rbc*L, *trn*LF and ITS genes were amplified with the PCR protocol^63^. Bidirectional sequences were generated by an ABI 3730 48 capillary sequencing machine using the BigDye Terminator v3.1 Cycle Sequencing Kit (Applied Biosystems, Foster City, CA, USA). Sequencing primers were the same as those used for the PCR. Newly generated sequences were assembled and edited with phyDE 0.9971. The new sequences were integrated into the *Lejeunea* dataset of Heinrichs et al.^27^ using BioEdit 5.0.9^64^. GenBank accession numbers and voucher details for newly sequenced taxa, which are represented by some 500 sequences, are listed in Supplementary Table S5. Only one specimen per species was selected to avoid biased age estimates^65^. As outgroups, we used five species of *Lepidolejeunea* following the approach of Heinrichs et al*.*^27^. Gene-specific alignments were performed using MAFFT 7^66^, and then concatenated into a supermatrix. Missing sequence stretches were coded as unknown, and ambiguously aligned regions as determined by eye were excluded from the dataset.

### Phylogenetic analyses

We reconstructed phylogenetic relationships using the supermatrix with ML and BI. We identified appropriate DNA partitions and corresponding substitution models, rate of invariable sites and gamma rate heterogeneity using PartitionFinder^67^ and the Akaike information criterion (AIC).

The ML analysis was conducted with RAxML-HPC 8.2.8^68^. Trees were generated by selecting 10 independent runs and the multi-parametric bootstrap option autoMRE, resulting in 300 bootstrap replicates to compute node support as bootstrap values at each node. Compatibility of the chloroplast and nuclear regions was explored by comparing the trees obtained from independent ML analyses for each region. The trees were visually compared to identify conflicting nodes with BV higher than 70%^69^, and an ML analysis was conducted with two chloroplast (*rbc*L, *trn*LF) partitions and a nuclear (ITS) partition.

BI was carried out with MrBayes 3.2.6^70^. The same dataset, nuclear substitution models and partitions as those in the ML analysis were used. Two simultaneous runs were performed with four Monte Carlo Markov chains (MCMCs) running for 10 million generations and sampling every 1,000th generation (resulting in 10,000 sampled trees and parameters). The runs were checked for convergence using the values of the potential reduction factor (PSRF; values close to 1.00) and effective sample size (ESS; values above 200) in Tracer 1.7^71^. The first 25% of the trees were discarded as burn-in. A 50% majority-rule consensus tree and posterior probability values were computed, and nodes were considered robust when PP ≥ 0.95^72^.

### Divergence times estimation

Molecular dating analyses were carried out with the supermatrix using BI as implemented in BEAST 1.8.2^73^ with 147 species, including outgroups. Nucleotide substitution models estimated with PartitionFinder were used, and all the parameters were estimated in BEAST. The fossil †*Microlejeunea nyiahae*^28^ provided a minimum age to constrain the *Lejeunea* clade (node 1 in Fig. 1). This fossil is preserved in amber (specimen number: AMNH-Tad-441-A) that was found in the Tadkeshwar Lignite Mine of Gujarat State, western India (N 21° 21.400, E 073° 04.532) dated from 52 Ma^74^. It represents the oldest crown-group fossil of Lejeuneaceae. The fossil calibration method used relies on constraining the minimum age of a taxon using the oldest known fossil^75,76^. The maximum age constraint was established as older than the fossil †*Microlejeunea nyiahae*^75,77^, where 95% of the prior space is up to 500 Ma older than the fossil. We are aware that using a single fossil may lead to biased molecular age estimates^78,79^. However, the Indian Cambay amber fossil used in this study allows substantially more precise and reliable dating than previously identified fossils of *Lejeunea* in Miocene Dominican amber. This is because the incompletely preserved sterile fossils in Miocene Dominican amber cannot be assigned confidently to any of the extant clades of *Lejeunea*. To avoid biasing the analysis with potentially unreliable fossils, we therefore did not use these additional fossils for node calibration. We also conducted divergence-time estimates without the fossil for comparison, i.e., based on gene-specific substitution rate calibration from the literature, where a plastid genome substitution rate of 5 × 10^–4^ subst./sites/myr^80,81^ was used for the three chloroplast regions and a substitution rate of 1.35 × 10^−3^ substitution/site/myr was used for the nucleus region^82^. We used a normal prior distribution in combination with the truncate option and upper and lower bounds of 0.4 and 8.3 × 10^−3^ substitution/site/myr, respectively^83^.

We conducted four dating analyses using different clock models. We used either the uncorrelated relaxed-clock model with a lognormal distribution of rates^84^ or the strict-clock model with a gamma distribution. Both relaxed-clock and strict-clock analyses were carried out with either a pure birth or a birth–death tree prior, with the latter considering incomplete sampling^85^. All the clock models were compared using Bayes factors calculated with path sampling^86^ and stepping-stone sampling^87^. For each model, we computed the marginal likelihood estimate^88,89^, allowing the computation of Bayes factors. The Bayesian inferences were run for 80 million generations, sampling every 8,000 generations. We used Tracer 1.7 to assess the convergence by examining the log files to check the ESS values for all parameters. The results were assumed reliable and as having good support when the ESS exceeded 200, indicating appropriate sampling. After a burn-in of 10% of trees, a maximum clade credibility tree was reconstructed to obtain mean node ages and 95% CI with TreeAnnotator 1.8.2. We used a normal distribution prior with a standard deviation of 1 Ma, and performed sensitivity analyses with a standard deviation of 5 Ma for comparison purpose.

### Ancestral areas estimation

We defined 9 areas of distribution of *Lejeunea* for the biogeographical analyses: A, the Neotropics; B, eastern North America; C, Europe; D, Africa (including Madagascar); E, continental Asia; F, tropical Asia; G, Australia-Pacific Islands; H, India; and I, western North America. The Rocky Mountains delimits the boundary between Eastern and Western North America, and the Tropic of Cancer (23° North) defines the boundary between continental and tropical Asia. The 9 biogeographical areas were defined based on the present-day distributions of the species and following Heinrichs et al.^27^, with possible changes in the disjunct ranges in the Northern Hemisphere to conform to our hypotheses. We obtained the species geographical distribution information from our data in the voucher specimen collection and the literature (Supplementary Table S6). All but 18 species in our 120-species dataset are restricted to one or two areas, e.g., species that are palaeotropical (*L. alata*, *L. exilis*, *L. papilionacea*, *L. pulchriflora*, *L. tuberculosa*), circumboreal (*L. cavifolia*), palaeotropical/pantropical (*L. anisophylla*, we are aware that this species is a synonym of Neotropical *L. adpressa* based on Gradstein^24^, but both species are maintained here for further analyses) and subcosmopolitan (*L. flava*). Ancestral ranges were reconstructed on the maximum clade credibility tree using the R package *BioGeoBEARS* 0.2.1^90^. We relied on the dispersal-extinction-cladogenesis (DEC) model^91^, which includes dispersal and extinction as free parameters and discarded all the other models, including the J parameter, which allows for jump dispersal (founder events). This is because of the statistical invalidity of the + J model of founder-event speciation and its comparison with the DEC model via model selection^92^. However, we are aware that conflicting results might occur with different ancestral-area estimations^90^; thus, we also used the DIVALIKE model for comparison (Supplementary Fig. S5). A time-stratified model with six time slides (Supplementary Table S7) that specified constraints on area connectivity through time was constructed, and these constraints represent the major geological changes thought to have affected the distribution of *Lejeunea*. We also tested an unconstrained model of DEC in which the dispersal multiplier matrices, the probabilities of all dispersal events were set to 1 and in all the analyses, the maximum range size was set to 9 areas (*L. flava* is distributed over the 9 areas).

### Environment-dependent diversification analyses

We assessed the effect of the palaeoenvironment on diversification rates using an environment-dependent model^34,93^. This model allows the testing of the potential effect that past environmental conditions had on the diversification of *Lejeunea* by evaluating whether speciation or extinction rates have varied with past environmental conditions, such as global variation in temperature. We used past temperature variations through the Cenozoic as inferred according to deep-sea oxygen^94^. We tested three models: (i) BVARDCST, the speciation rate varies exponentially with temperature, and the extinction rate is constant; (ii) BCSTDVAR, the speciation rate is constant, and the extinction rate varies exponentially with temperature; and (iii) BVARDVAR, both the speciation and extinction rates vary exponentially with temperature. Subsequently, we repeated the tests of these three models with linear dependence on temperature. We computed the corrected AIC (AICc) for each diversification model to select the best-fit temperature-dependent model. Analyses were run over 500 trees randomly taken from the Bayesian dating analysis.

### Trait-dependent diversification analyses

The binary-state speciation and extinction (BiSSE) model^95^ was used to assess whether the sexual systems of *Lejeunea* (monoicous or dioicous) have a significant influence on the diversification rates. The BiSSE model analyses were carried out using the R package *diversitree* 0.9–3^96^. A total of seven models were evaluated, and for the best-fit model, an MCMC analysis was performed to compute the 95% CI of the parameters. We used the exponential prior 1/(2*r*) and began the chain with the parameters obtained by maximum likelihood. We ran 20,000 steps of the MCMC and applied a burn-in of 2,000 steps. SSE models tend to have a high type I error bias^97–99^. To test the robustness of our diversification results, we first estimated the difference in fit (ΔAIC) between the best BiSSE model and a null model in which rates do not vary with traits, and compared this with the difference between the same models as estimated from simulated datasets. Second, we performed the two-state character-independent diversification (CID-2) model^98,99^. This CID-2 model explicitly assumes that the evolution of a binary character is independent of the diversification process without forcing the diversification process to be constant across the entire tree. CID-2 contains four diversification parameters that account for trait-dependent diversification solely on the two states of an unobserved, hidden trait. We used the R package *hisse* 1.9.1^98^ to set up a model where the diversification process is independent from the observed states (0 or 1) of the focal trait. BiSSE and HiSSE analyses were also run over 500 trees randomly taken from the Bayesian dating analysis.

## Supplementary information

Supplementary Information

## Data Availability

DNA sequences each species of *Lejeunea* used in the present study are deposited in GenBank.
