# Supplementary material for: An ancient tropical origin, dispersals via land bridges and Miocene diversification explain the subcosmopolitan disjunctions of the liverwort genus Lejeunea
Source: Sci Rep. 2020 Aug 24;10:14123. doi: 10.1038/s41598-020-71039-1 (PMC7445168; doi:10.1038/s41598-020-71039-1)

**Supplementary Material**

# An ancient tropical origin, dispersals via land bridges and Miocene diversification explain the subcosmopolitan disjunctions of the liverwort genus *Lejeunea*

*Gaik Ee Lee, Fabien L. Condamine, Julia Bechteler, Oscar Alejandro Pérez-Escobar, Armin Scheben, Alfons Schäfer-Verwimp, Tamás Pócs & Jochen Heinrichs*

__Contents__

**Supplementary Table S1**
Divergence times estimation with a normal distribution prior and a standard deviation of 5 Ma.

**Supplementary Table S2**
Comparison of biogeographical models in BioGeoBEARS. lnL: log likelihood, *d*: dispersal rate, *e*: extinction rate, AIC: Akaike information criterion.

**Supplementary Table S3**
Result from the paleoenvironment-dependent diversification models.

**Supplementary Table S4**
Result from the BiSSE analyses exploring the interdependence of diversification rate on sexual system (dioicous or monoicous).

**Supplementary Table S5**
Taxa used in this study with information about the origin of the studied material, vouchers, as well as GenBank accession numbers and new sequences (bold face).

**Supplementary Table S6**
The nine defined areas of distribution of *Lejeunea* for the biogeographical analyses.

**Supplementary Table S7**
The time-stratified biogeographic model that specified constraints on area connections used for DEC analyses.

**Supplementary Figure S1**
Maximum Likelihood (ML) phylogeny of *Lejeunea* based on three markers from nuclear and plastid DNA. ML bootstrap probabilities ≥ 50 are shown at branches; a star indicates a

Posterior Probability ≥ 0.95.

**Supplementary Figure S2**
Reconstruction of the biogeographical history for *Lejeunea* using an unconstrained DEC model as inferred from BioGeoBEARS.

**Supplementary Figure S3**
The pie chart from the reconstruction of the biogeographical history for *Lejeunea* using a time-stratified DEC model as inferred from BioGeoBEARS.

**Supplementary Figure S4**
Robustness of BiSSE models with simulation test. For the simulation, the difference in fit (ΔAIC) between the best BiSSE model and the null model is shown with the red vertical line for real data, and in gray for simulated datasets.

**Supplementary Figure S5**
Reconstruction of the biogeographical history for *Lejeunea* using a time-stratified DIVALIKE model as inferred from BioGeoBEARS.

**Table S1** Divergence time estimates of main clades of *Lejeunea* obtained from four analyses in BEAST with a normal distribution prior and a standard deviation of 5 Ma. RC, Birth-Death: Relaxed clock with Birth-Death tree including incomplete sampling; RC, Yule: Relaxed clock using Yule parameter; SC, Birth-Death: Strict clock with Birth-Death tree including incomplete sampling; SC, Yule: Strict clock using Yule parameter.

| Node | RC, birthdeath | RC, Yule | SC, birthdeath | SC, Yule |
|---|---|---|---|---|
| 1. *Lejeunea* stem | 54.95 (46.34-63.80) | 55.01 (45.04-62.13) | 53.01 (44.18-61.36) | 53.71 (43.41-60.10) |
| 2. *Lejeunea* crown | 45.80 (37.65-54.66) | 45.50 (34.70-53.91) | 42.66 (35.21-50.94) | 43.52 (33.06-51.78) |
| 3. *Lejeunea* clade III crown | 40.65 (33.09-48.10) | 39.74 (30.07-46.16) | 37.45 (30.79-43.95) | 37.64 (29.28-42.76) |
| 4. *Lejeunea* clade IV crown | 35.29 (28.65-42.23) | 34.28 (25.93-39.64) | 32.95 (27.35-38.64) | 33.19 (25.89-37.20) |
| 5. *Lejeunea* clade I crown | 34.53 (27.49-41.75) | 33.52 (24.91-40.15) | 30.87 (25.02-36.61) | 31.19 (23.37-35.32) |
| 6. *Lejeunea* clade II crown | 33.03 (24.64-36.94) | 32.07 (23.48-37.06) | 29.38 (24.03-34.35) | 29.64 (22.88-33.46) |
| 7. *Lejeunea* clade V crown | 30.92 (23.88-35.82) | 29.94 (22.39-34.60) | 27.91 (23.05-32.85) | 28.26 (21.61-31.56) |
| 8. Lejeunea clade VI crown | 29.83 (21.59-32.37) | 28.82 (21.47-33.21) | 24.54 (20.17-28.76) | 26.96 (20.78-30.25) |

**Table S2** Comparison of biogeographical models in BioGeoBEARS. lnL: log likelihood, *d*: dispersal rate, *e*: extinction rate, AIC: Akaike information criterion.

| Model | lnL | No. of parameters | *d* | *e* | AIC |
|---|---|---|---|---|---|
| Unconstrained* | | | | | |
| DEC | -494.5 | 2 | 0.0053 | 0.0002 | 993.1 |
| DIVALIKE | -518.5 | 2 | 0.0064 | 0.0000 | 1041 |
| BAYAREALIKE | -518.0 | 2 | 0.0036 | 0.0330 | 1040 |
| | | | | | |
| Constrained** | | | | | |
| DEC | -798.4 | 2 | 0.1483 | 0.0583 | 1276 |
| DIVALIKE | -798.4 | 2 | 0.1365 | 0.0492 | 1274 |
| BAYAREALIKE | -827.1 | 2 | 0.1004 | 0.0682 | 1303 |

*with the default setting in which the dispersal multiplier matrices were set to 1

**a time-stratified model with time slices that specified constraints on area connections

**Table S3** Result from paleoenvironmental-dependent diversification models based 500 posterior trees. df, degree of freedom; logL, log likelihood; λ, speciation rate; α, rate of variation of the speciation based on the paleoenvironmental variable; μ, extinction rate; β, rate of variation of the extinction based on the paleoenvironmental variable. Bold and yellow highlighted lines indicate the best model of each approach. The ΔAIC are used for comparison in all the models and for selection of the best model.

| Models | Mode of dependence | df | logL | AICc | ΔAIC | λ | α | μ | β |
|---|---|---|---|---|---|---|---|---|---|
| λ constant and μ constant | - | 2 | 441.872 ±0.548 | 887.845 ±1.097 | null model | 0.1192 ±0.0007 | - | 0.0066 ±0.0006 | - |
| λTemp. and μ constant | exponential | 3 | 441.546 ±0.557 | 889.297 ±1.115 | 2.121 | 0.1121 ±0.0009 | 0.0143 ±0.0014 | 0.0105 ±0.0012 | - |
| λ constant and μTemp. | exponential | 3 | 441.725 ±0.549 | 889.657 ±1.098 | 2.481 | 0.1189 ±0.0006 | - | 0.0067 ±0.0012 | 0.1072 ±0.0110 |
| λTemp. and μTemp. | exponential | 4 | 441.387 ±0.556 | 891.119 ±1.112 | 3.943 | 0.1061 ±0.0010 | 0.0311 ±0.0023 | 0.0106 ±0.0014 | 0.0495 ±0.0047 |
| **λTemp.** | **linear** | **2** | **441.537 ±0.563** | **887.176 ±1.126** | **0** | **0.1070 ±0.0011** | **0.0013 ±0.0002** | **-** | **-** |
| λTemp. and μ constant | linear | 3 | 441.467 ±0.561 | 889.140 ±1.123 | 1.964 | 0.1071 ±0.0012 | 0.0028 ±0.0002 | 0.0133 ±0.0015 | - |
| λ constant and μTemp. | linear | 3 | 441.491 ±0.549 | 889.186 ±1.099 | 2.009 | 0.1321 ±0.0010 | - | 0.0747 ±0.0039 | 0.0102 ±0.0006 |
| λTemp. and μTemp. | linear | 4 | 440.288 ±0.595 | 888.921 ±0.001 | 1.747 | 0.0892 ±0.0018 | 0.0163 ±0.0008 | 0.1254 ±0.0043 | 0.0182 ±0.0011 |

**Table S4** Result from the BiSSE analyses exploring the interdependence of diversification rate on sexual system (dioicous or monoicous) based on 500 posterior trees. M, monoicous; D, dioicous; df, degree of freedom; logL, log likelihood; $\lambda$, speciation rate; $\mu$, extinction rate; q, character state transition rate. Bold and yellow highlighted lines indicate the best model. The ΔAIC are used for comparison in all the models and for selection of the best model.

| Model | df | logL | AICc | ΔAIC | $\lambda_M$ | $\lambda_D$ | $\mu_M$ | $\mu_D$ | $q_{M\text{-}D}$ | $q_{D\text{-}M}$ |
|---|---|---|---|---|---|---|---|---|---|---|
| $\lambda_M = \lambda_D$, $\mu_M = \mu_D$, $q_{M\text{-}D} = q_{D\text{-}M}$ | 3 | -513.111 ±0.647 | 1032.426 ±1.295 | 1.958 | - | 0.1096 ±0.0005 | - | 0.0022 ±0.0003 | - | 0.0204 ±0.0001 |
| **$\lambda_M \neq \lambda_D$, $\mu_M = \mu_D$, $q_{M\text{-}D} = q_{D\text{-}M}$** | **4** | **-511.061 ±0.666** | **1030.468 ±1.332** | **0** | **0.1298 ±0.0008** | **0.0941 ±0.0006** | - | **0.0005 ±0.0001** | - | **0.0204 ±0.0005** |
| $\lambda_M = \lambda_D$, $\mu_M \neq \mu_D$, $q_{M\text{-}D} = q_{D\text{-}M}$ | 4 | -512.615 ±0.648 | 1033.575 ±1.297 | 3.107 | - | 0.1159 ±0.0006 | 0.0004 ±0.0001 | 0.0198 ±0.0006 | - | 0.0192 ±0.0002 |
| $\lambda_M = \lambda_D$, $\mu_M = \mu_D$, $q_{M\text{-}D} \neq q_{D\text{-}M}$ | 4 | -512.446 ±0.651 | 1033.237 ±1.301 | 2.769 | - | 0.1097 ±0.0005 | - | 0.0021 ±0.0003 | 0.0150 ±0.0002 | 0.0244 ±0.0002 |
| $\lambda_M \neq \lambda_D$, $\mu_M \neq \mu_D$, $q_{M\text{-}D} = q_{D\text{-}M}$ | 5 | -510.972 ±0.664 | 1032.466 ±1.328 | 1.998 | 0.1345 ±0.0010 | 0.0945 ±0.0006 | 0.0081 ±0.0008 | 0.0007 ±0.0002 | - | 0.0007 ±0.0002 |
| $\lambda_M \neq \lambda_D$, $\mu_M = \mu_D$, $q_{M\text{-}D} \neq q_{D\text{-}M}$ | 5 | -510.531 ±0.668 | 1031.584 ±1.337 | 1.331 | 0.1289 ±0.0009 | 0.0956 ±0.0006 | - | 0.0008 ±0.0002 | 0.0157 ±0.0004 | 0.0212 ±0.0003 |
| $\lambda_M = \lambda_D$, $\mu_M \neq \mu_D$, $q_{M\text{-}D} \neq q_{D\text{-}M}$ | 5 | -512.139 ±0.651 | 1034.799 ±1.303 | 4.331 | - | 0.1145 ±0.0006 | 0.0015 ±0.0002 | 0.0145 ±0.0007 | 0.0155 ±0.0002 | 0.0223 ±0.0002 |
| $\lambda_M \neq \lambda_D$, $\mu_M \neq \mu_D$, $q_{M\text{-}D} \neq q_{D\text{-}M}$ | 6 | -510.279 ±0.667 | 1033.295 ±1.335 | 2.827 | 0.1481 ±0.0015 | 0.0969 ±0.0006 | 0.0308 ±0.0017 | 0.0002 ±0.0001 | 0.0143 ±0.0003 | 0.0234 ±0.0003 |

**Table S5** Taxa used in the present study with information about the origin of the studied material, vouchers, as well as GenBank accession numbers is included. New sequences in bold face.

| Taxon | Origin | Collector | GenBank Accession No. | | |
|---|---|---|---|---|---|
| | | | *rbc*L | *trn*LF | ITS |
| *Harpalejeunea. grandis* Grolle & M.E.Reiner | Colombia | Cleef 6450 (GOET) | KC313144 | KC313184 | KC313106 |
| *H. grandistipula* R.M.Schust. | Ecuador | Schäfer-Verwimp et al. 24163/B (GOET) | KC313145 | KC313185 | KC313107 |
| *H. marginalis* (Hook.f. & Taylor) Steph. | Chile | Schäfer-Verwimp & Verwimp 8082 (GOET) | KC313147 | KC313187 | KC313109 |
| *H. molleri* (Steph.) Grolle | Azores | Schäfer-Verwimp & Verwimp 29334 (GOET) | KC313148 | KC313188 | KC313110 |
| *H. stricta* (Lindenb. & Gottsche) Steph. | Panama | Schäfer-Verwimp & Verwimp 31036 (GOET) | KX113507 | KX113483 | KX113495 |
| ***H. uncinata* Steph.** | **Panama** | **Schäfer-Verwimp & Verwimp 34146 (M)** | **MN822945** | **MN823005** | **MN818965** |
| *Lejeunea acuta* Mitt. | Kenya | Chuah-Petiot Mb 22 (JE) | KF556384 | KF556134 | KF555917 |
| *L. adpressa* Nees | Dominican Rep. | Schäfer-Verwimp & Verwimp 26931/B (GOET) | KF556386 | KF556136 | KF555919 |
| *L. alata* Gottsche | Malaysia | Schäfer-Verwimp & Verwimp 18912 (GOET) | ----------- | KF556140 | KF555922 |
| ***L. alata* var. *patriciae* Pócs** | **Samoa** | **Bartlett 32262b (JE)** | **MN822951** | **MN823014** | **MN818974** |
| ***L. albescens* (Steph.) Mizut.** | **Sabah, Borneo** | **G.E.Lee 1533 (UKMB)** | **MN822952** | **MN823015** | **MN818975** |

| | | | | | |
|---|---|---|---|---|---|
| *L. amaniensis* E.W.Jones | Kenya | Malombe & Chituyi 5006/Si.1aS5 (EGR) | KF556392 | KF556143 | KF556603 |
| ***L. anisophylla* Nees & Mont.** | **Sabah, Borneo** | **G.E.Lee 2145 (UKMB)** | **MN822953** | **MN823016** | **MN818976** |
| ***L. apiculata* Sande Lac.** | **Peninsular Malaysia** | **Pócs & Pócs 1003/AC (EGR)** | **MN823003** | **MN823017** | **MN818977** |
| *L. aquatica* Horik. | Japan | Higuchi 1021 (JE) | ----------- | KF556155 | KF555933 |
| *L. asperrima* Spruce | Panama | Schäfer-Verwimp & Verwimp 30817 (GOET) | KF556402 | KF556157 | KF555935 |
| *L. asperula* (Steph.) Mizut. | Papua New Guinea | Streimann 40815 (JE) | ----------- | KF556156 | KF555934 |
| *L. bermudiana* (A.Evans) R.M.Schust. | USA | Shaw 14939 (DUKE) | ----------- | KF556158 | KF555936 |
| *L. boryana* Mont. | French Guiana | Holz FG 00-0103 (GOET) | KF556405 | KF556159 | KF555938 |
| ***L. brenanii* E.W.Jones** | **São Tomé** | **Shevock 39785 (M)** | **MN822954** | **MN823018** | **MN818978** |
| *L. cancellata* Nees & Mont. | Costa Rica | Schäfer-Verwimp & Holz SV/H-0507/C (GOET) | KF556409 | KF556164 | KF555942 |
| *L. capensis* Gottsche | Brazil | Schäfer-Verwimp & Verwimp 15057 (GOET) | ----------- | KF556167 | KF555943 |
| ***L. caracensis* Lindenb.** | **Venezuela** | **Picón et al. 00227/CD (EGR)** | **MN822955** | **MN823019** | **MN818979** |
| *L. catinulifera* Spruce | Ecuador | Wilson et al. 04-01 (GOET) | DQ983687 | DQ987432 | DQ987328 |
| *L. cavifolia* (Ehrh.) Lindb. | Germany | Heinrichs 3695 (GOET) | AY548102 | DQ238581 | DQ987259 |
| *L. cerina* (Lehm. & Lindenb.) Gottsche et al. | Costa Rica | Schäfer-Verwimp & Holz SV/H-0471 (GOET) | KF556425 | KF556180 | KF555955 |

| | | | | | |
|---|---|---|---|---|---|
| ***L. cocoes* Mitt.** | **Peninsular Malaysia** | **G.E.Lee 2339 (UKMB)** | **MN822957** | **MN823021** | **MN818963 (ITS1)** |
| *L. colensoana* (Steph.) M.A.M.Renner | New Zealand | Renner 300140 (AK) | ----------- | JF308578 | JF308549 |
| ***L. compacta* (Steph.) Steph.** | **China** | **Long 18826 (E)** | **MN822956** | **MN823020** | **MN818980** |
| *L. compressiuscula* (Steph.) G.E.Lee & Heinrich | Indonesia | Schäfer-Verwimp & Verwimp 24923/E (GOET) | KF556519 | KF556290 | KF556050 |
| ***L. conformis* Nees & Mont.** | **São Tomé** | **Shevock 34483 (EGR)** | **MN822958** | **MN823022** | **MN818981** |
| *L. controversa* Gottsche | French Guiana | Hartmann et al. 04-033 (GOET) | KF556432 | KF556189 | KF555964 |
| ***L. corynantha* Spruce** | **Dominica** | **Schäfer-Verwimp & Verwimp 17961/A (GOET)** | **MN995825** | **MN995826** | **MN990423** |
| *L. cristulata* (Steph.) M.E.Reiner & Goda | Brazil | Giancotti 17 (JE) | ----------- | KF556193 | KF555966 |
| *L. curviloba* Steph. | Bhutan | Long 10611 (JE) | ----------- | KF556195 | KF555967 |
| ***L. cyathophora* Mitt.** | **Costa Rica** | **Schäfer-Verwimp & Holz SV/H- 229 (M)** | **MN822959** | **MN823023** | **MN818982** |
| *L. debilis* (Lehm. & Lindenb.) Nees & Mont. | Costa Rica, La Gamba | Schluder 7 (GOET) | KF556437 | KF556197 | KF555969 |
| *L. deplanata* Nees | Ecuador, Pichincha | Schäfer-Verwimp et al. 24502/C (GOET) | KF556439 | KF556199 | KF555971 |
| ***L. dimorpha* Kodama** | **Peninsular Malaysia** | **G.E.Lee 2211 (UKMB)** | **MN822960** | **MN823024** | **MN818983** |
| ***L. dipterocarpa* E.W.Jones** | **Equatorial Guinea** | **Müller B316 (EGR)** | **MN822961** | **MN823025** | **MN818984** |
| ***L. dipterota* (Eifrig) G.E.Lee** | **Sabah, Borneo** | **G.E.Lee 1802 (UKMB)** | **MN822962** | **MN823026** | **MN818985** |

| | | | | | |
|---|---|---|---|---|---|
| ***L. discreta* Lindenb.** | **Peninsular Malaysia** | **G.E.Lee 2328 (UKMB)** | **MN822963** | **MN823027** | **MN818986** |
| *L. drehwaldii* Heinrichs & Schäf.-Verw. | Peru | Drehwald 4384 (JE) | KF556445 | KF556207 | KF555978 |
| *L. eckloniana* Lindenb. | Madeira | Stech 04-433 (L) | KF556447 | KF556211 | KF555983 |
| ***L. eifrigii* Mizut.** | **Peninsular Malaysia** | **G.E.Lee 2334 (UKMB)** | **MN822964** | **MN823028** | **MN818987** |
| *L. exilis* (Reinw. et al.) Grolle | Indonesia | Schäfer-Verwimp & Verwimp 25231 (GOET) | KF556449 | KF556213 | KF555985 |
| *L. flava* (Sw.) Nees | Dominican Rep. | Schäfer-Verwimp & Verwimp 26855/B (GOET) | KF556479 | KF556243 | KF556009 |
| ***L. fleischeri* (Steph.) Mizut.** | **Indonesia** | **Schäfer-Verwimp & Verwimp 24809/A (GOET)** | **MN822965** | **MN823029** | **MN818988** |
| ***L. glaucescens* Gottsche** | **Brazil** | **Schäfer-Verwimp & Verwimp 9724 (M)** | **MN822966** | **MN823060** | **MN818989** |
| ***L. globosiflora* (Steph.) Steph.** | **Chile** | **Gradstein 12418 (GOET)** | **MN822967** | **MN823030** | **MN818962/ MN818960** |
| ***L. gradsteinii* G.E.Lee et al.** | **Sabah, Borneo** | **G.E.Lee 1885 (UKMB)** | **MN822968** | **MN823031** | **MN818990** |
| *L. grossitexta* (Steph.) M.E.Reiner & Goda | Panama | Schäfer-Verwimp & Verwimp 31000 (GOET) | KF556491 | KF556256 | KF556020 |
| *L. helmsiana* Steph. | New Zealand | Renner 300069 (AK) | ----------- | JF308569 | JF308540 |
| ***L. hepaticola* Steph.** | **Kenya** | **Pócs & Pócs 04004/K (EGR)** | **MN822969** | **MN823032** | **MN818991** |
| *L. hibernica* Grolle | Ireland | Long 11743 (JE) | ----------- | KF556257 | KF556021 |
| *L. holtii* Spruce | Madeira | Drehwald & Drehwald 3719 (GOET) | KF556492 | KF556258 | KF556022 |

| | | | | | |
|---|---|---|---|---|---|
| ***L. ibadana* A.J.Harr & E.W.Jones** | **Príncipe Isl.** | **Shevock 42309 (EGR)** | **MN822970** | **MN823033** | **MN818992** |
| *L. intricata* J.B.Jack & Steph. | Ecuador | Schäfer-Verwimp & Nebel 33217 (GOET) | ----------- | ----------- | KF556023 |
| *L. isocalycina* (Nees) Spruce | Brazil | Costa & Gradstein 3720 (GOET) | KF556496 | KF556262 | KF556027 |
| *L. isophylla* E.W.Jones | Madagascar | Lübenau 21 (EGR) | KF556497 | KF556263 | KF556028 |
| *L. japonica* Mitt. | Japan | Mizutani 15618 (L) | KF556499 | KF556265 | KF556030 |
| ***L. kinabalensis* Mizut.** | **Sabah, Borneo** | **G.E.Lee 2138 (UKMB)** | **MN822971** | **MN823034** | **MN818993** |
| ***L. konosensis* Mizut.** | **China** | **Long & Shevock 37403 (E)** | **MN822972** | **MN823035** | **MN818994** |
| ***L. kuerschneriana* Pócs** | **Kenya** | **Chuah et al. 3017 (EGR)** | **MN822973** | **MN823036** | **MN818995** |
| *L. laeta* (Lehm. & Lindenb.) Gottsche et al. | Ecuador | Schäfer-Verwimp et al. 24412 (GOET) | ----------- | KF556267 | KF556032 |
| *L. laetevirens* Nees & Mont. | Dominican Rep. | Schäfer-Verwimp & Verwimp 27079 (GOET) | KF556508 | KF556275 | KF556037 |
| *L. lamacerina* (Steph.) Schiffn. | Azores | Schäfer-Verwimp & Verwimp 29394 (GOET) | KF556510 | KF556279 | KF556041 |
| ***L. leratii* (Steph.) Mizut.** | **New Caledonia** | **Larrín 35964 (EGR)** | **MN822974** | **MN823062** | **MN818996** |
| *L. lomana* E.W.Jones | Réunion | Pócs 08064/L (EGR) | KF556388 | KF556138 | KF555921 |
| ***L. lumbricoides* (Nees) Nees** | **Peninsular Malaysia** | **G.E.Lee 1429 (UKMB)** | **MN822975** | **MN823037** | **MN818997** |
| ***L. micholitzii* Mizut.** | **Sri Lanka** | **Schäfer-Verwimp & Verwimp 16374 (M)** | **MN822976** | **MN823063** | **MN818998** |

| | | | | | |
|---|---|---|---|---|---|
| *L. microloba* Taylor | Fiji Isls. | Pócs 08013/Y (EGR) | KF556520 | KF556291 | KF556051 |
| ***L. mimula* Hürl.** | **Sabah, Borneo** | **G.E.Lee 1749 (UKMB)** | **MN822977** | **MN823038** | **MN818999** |
| ***L. mizutanii* Grolle** | **New Caledonia** | **von Konrat s.n. (EGR)** | **MN822978** | **MN823039** | **MN819000** |
| *L. monimiae* (Steph.) Steph. | Ecuador | Schäfer-Verwimp & Preussing 23226/A (GOET) | KF556526 | KF556298 | KF556055 |
| *L. multidentata* M.E.Reiner & Mustelier | Dominican Rep. | Pócs & Pócs 03157/A (EGR) | KF556527 | KF556299 | KF556056 |
| *L. neelgherriana* Gottsche | Japan | Higuchi 0791954 (L) | ----------- | KF556301 | KF556058 |
| *L. nepalensis* (Steph.) H.A.Mill. et al. | Nepal | Long 17250 (JE) | ----------- | KF556302 | KF556059 |
| ***L. obscura* Mitt.** | **Indonesia** | **Schäfer-Verwimp & Verwimp 24957/C (M)** | **MN822980** | **MN823041** | **MN819002** |
| *L. obtusangula* Spruce | Bolivia | Krömer 869 (GOET) | KF556532 | KF556307 | KF556063 |
| ***L. obtusata* Gottsche** | **Kenya** | **Pócs et al. 04042/G (EGR)** | **MN822979** | **MN823040** | **MN819001** |
| *L. oligoclada* Spruce | Brazil | Schäfer-Verwimp & Verwimp 13590 (GOET) | KF556533 | KF556308 | KF556064 |
| *L. oracola* M.A.M.Renner | New Zealand | Renner 300078 (AK) | ----------- | JF308571 | JF308542 |
| *L. osculatiana* De Not. | Panama | Schäfer-Verwimp & Verwimp 30958 (GOET) | KF556538 | KF556314 | KF556631 |
| *L. pallescens* Mitt. | Ecuador | Schäfer-Verwimp 32731 (GOET) | KF556540 | ----------- | KF556069 |
| *L. parva* (S.Hatt.) Mizut. | Japan | Mizutani 15293 (L) | KF556542 | KF556318 | KF556072 |

| | | | | | |
|---|---|---|---|---|---|
| ***L. papilionacea* Prantl.** | **São Tomé** | **Shevock 42335 (EGR)** | **MN822981** | **MN823061** | **MN818961 (ITS2)** |
| ***L. patersonii* (Steph.) Steph.** | **Thailand** | **G.E.Lee 2525 (UKMB)** | **MN822982** | **MN823042** | **MN819003** |
| ***L. patriciae* Schäf.-Verw.** | **Peninsular Malaysia** | **Pócs et al. 13161/2 (EGR)** | **MN822983** | **MN823043** | **MN819004** |
| *L. paucidentata* (Steph.) Grolle | Cuba | Pócs & Caluff 9199/CL (JE) | ----------- | KF556321 | KF556075 |
| ***L. pectinella* Mizut.** | **Sabah, Borneo** | **Lee 1672 (UKMB)** | **MN822984** | **MN823044** | **MN819005** |
| *L. phyllobola* Nees & Mont. | Ecuador | Noeske et al. 204 (GOET) | KF556600 | KF556322 | KF556076 |
| *L. pterigonia* (Lehm. & Lindenb.) Mont. | Bolivia | Churchill et al. 21851 (GOET) | KF556546 | KF556325 | KF556078 |
| ***L. ptosimophylla* C.Massal.** | **Argentina** | **Vinocur MER502 (GOET)** | ----------- | **MN823045** | **MN818964 (ITS1)** |
| *L. puiggariana* Steph. | Dominican Rep. | Schäfer-Verwimp & Verwimp 27016/A (GOET) | KF556550 | KF556329 | KF556082 |
| *L. pulchriflora* (Pearson) G.E.Lee et al. | Tanzania | Pócs 89126/M (EGR) | KT897948 | KT897953 | KT897943 |
| *L. pulverulenta* (Gottsche ex Steph.) M.E.Reiner | Bolivia | Reiner-Drehwald & Drehwald 4517 (GOET) | KF556552 | KF556331 | KF556084 |
| *L. ramosissima* Steph. | São Tomé and Príncipe | Shevock 34348A (EGR) | KF556554 | KF556333 | KF556086 |
| *L. ramulosa* (Herzog) R.M.Schust. | Costa Rica | Schäfer-Verwimp & Holz SV/H-0229/A (GOET) | KF556555 | KF556335 | KF556088 |
| *L. reflexistipula* (Lehm. & Lindenb.) Gottsche et al. | Ecuador (III) | Schäfer-Verwimp & Nebel 33162 (GOET) | KF556559 | KF556340 | KF556092 |
| ***L. reinerae* Ilk.-Borg.** | **Fiji Isl.** | **Pócs & Pócs 03261/AC (EGR)** | **MN822985** | **MN823046** | **MN819006** |

| | | | | | |
|---|---|---|---|---|---|
| *L. rotundifolia* Mitt. | Costa Rica, Cartago | Schäfer-Verwimp & Holz SV/H-0378 (GOET) | KF556567 | KF556348 | KF556099 |
| *L. ruthii* (A.Evans) R.M.Schust. | USA, Tennessee | Zartman 681 (DUKE) | KF556569 | ----------- | KF556634 |
| ***L. sikorae* (Steph.) Steph.** | **Madagascar** | **Pócs & Szabó 9878/EZ (EGR)** | **MN822986** | **MN823047** | **MN819007** |
| ***L. soae* R.L.Zhu et al.** | **China** | **Long 24473b (E)** | **MN822987** | **MN823048** | **MN819008** |
| ***L. sordida* (Nees) Nees** | **Papua New Guinea** | **Kumei 15 (JE)** | **MN822988** | **MN823049** | **MN819009** |
| *L. sporadica* Besch. & Spruce | Panama | Schäfer-Verwimp & Verwimp 31033 (GOET) | KF556583 | ----------- | KF556117 |
| ***L. stenodentata* M.A.M.Renner & Pócs** | **Papua New Guinea** | **Streimann 41784 (JE)** | **MN822989** | **MN823050** | **MN819010** |
| ***L. stephaniana* Mizut.** | **Sabah, Borneo** | **G.E.Lee 1515 (UKMB)** | **MN822990** | ----------- | **MN819011** |
| ***L. stevensiana* (Steph.) Mizut.** | **China** | **Long 37118 (E)** | **MN822991** | **MN823051** | **MN819012** |
| ***L. subolivacea* Mizut.** | **Bangladesh** | **Long 28096 (E)** | **MN822992** | **MN823052** | **MN819013** |
| ***L. subplana* (Steph.) C.Bastos** | **Brazil** | **Schäfer-Verwimp & Verwimp 6953/B (M)** | **MN822993** | **MN823053** | **MN819014** |
| *L. subspathulata* Spruce | Dominican Rep. | Gradstein 6643 (GOET) | KF556585 | KF556367 | KF556119 |
| ***L. sulphurea* (Lehm. & Lindenb.) Spruce** | **Guadeloupe** | **Schäfer-Verwimp 22453 (GOET)** | **MN822994** | **MN823054** | **MN819015** |
| *L. tapajosensis* Spruce | Ecuador | Nöske et al. 204 (GOET) | KF556589 | KF556371 | KF556122 |
| *L. tasmanica* Gottsche et al. | New Zealand | Renner 872054 (NSW) | ----------- | JF308581 | JF308552 |

| | | | | | |
|---|---|---|---|---|---|
| ***L. terricola*** **Spruce** | **Argentina** | **Reiner MER1148 (M)** | **MN822995** | **MN823055** | **MN819016** |
| ***L. thallophora*** **(Eifrig) Gradst.** | **Indonesia** | **Abrahamczyk 05285 (JE)** | **MN822996** | **MN823056** | **MN819017** |
| *L. topoensis* Gradst. & M.E.Reiner | Ecuador | Gradstein & Jost 10063 (GOET) | DQ983712 | DQ987416 | DQ987312 |
| *L. trinitensis* Lindenb. & Gottsche | Brazil | Vital 10.168 (JE) | KF556594 | KF556376 | KF556127 |
| ***L. tuberculosa*** **Steph.** | **São Tomé and Príncipe** | **Shevock 42453 (EGR)** | **MN822997** | **MN823057** | **MN819018** |
| *L. tumida* Mitt. | New Zealand | Renner 299949 (AK) | ----------- | JF308556 | JF308527 |
| *L. umbilicata* (Nees) Nees et al. | Indonesia | Schäfer-Verwimp & Verwimp 16954 (GOET) | KF556597 | KF556379 | KF556130 |
| ***L. urbanii*** **Steph.** | **Dominican Rep.** | **Schäfer-Verwimp & Verwimp 27006 (GOET)** | **MN822998** | **MN823058** | **MN819019** |
| ***L. utriculata*** **(Steph.) Mizut.** | **Peninsular Malaysia** | **Schäfer-Verwimp & Verwimp 18900/C (M)** | **MN822999** | **MN823059** | **MN819020** |
| ***L. wightii*** **Lindenb.** | **Thailand** | **Schäfer-Verwimp & Verwimp 23844 (M)** | **MN823000** | **MN823004** | **MN819021** |
| *Microlejeunea africana* Steph. | São Tomé and Príncipe | Shevock 34576B (GOET) | KC313151 | KC313191 | KC313113 |
| ***M. ankasica*** **E.W.Jones** | **São Tomé and Príncipe** | **Shevock 42048 (EGR)** | **MN823002** | **MN823006** | **MN818966** |
| ***M. bullata*** **(Taylor) Steph.** | **Brazil** | **Peralta et al. 14921 (M)** | **MN823001** | **MN823007** | **MN818967** |
| *M. capillaris* (Gottsche) Steph. | Costa Rica | Schäfer-Verwimp & Holz SV/H-0489/B (JE) | KC313152 | KC313192 | KC313114 |
| *M. colombiana* Bischl. | Dominican Rep. | Schäfer-Verwimp & Verwimp 26614/A (JE) | KC313153 | KC313193 | KC313079 |

| | | | | | |
|---|---|---|---|---|---|
| ***M. cystifera* Herzog** | **Brazil** | **Peralta et al. 15764 (M)** | **MN822946** | **MN823008** | **MN818968** |
| *M. filicuspis* (Steph.) Heinrichs et al. | Fiji Isls. | Pócs & Pócs 03304/A (EGR) | KC313138 | KC313178 | KC313100 |
| *M. fischeri* (Tixier) Heinrichs et al. | Uganda | Pócs & Lye 97141/T (EGR) | KC313140 | KC313180 | KC313102 |
| ***M. globosa* (Spruce) Steph.** | **Brazil** | **Schäfer-Verwimp 34053 (M)** | **MN822947** | **MN823009** | **MN818969** |
| ***M. kamerunensis* Steph.** | **Equatorial Guinea** | **Müller B999/D (M)** | **MN822948** | **MN823010** | **MN818970** |
| *M. latitans* (Hook.f & Taylor) Heinrichs et al. | New Zealand | Schäfer-Verwimp & Verwimp 13869 (JE) | KC313146 | KC313186 | KC313108 |
| ***M. nyandaruensis* Pócs** | **Kenya** | **Pócs 02031RB (EGR)** | **MN822949** | **MN823011** | **MN818971** |
| ***M. punctiformis* (Taylor) Steph.** | **Singapore** | **Pócs & Tan 99203/C (EGR)** | **MN822944** | **MN823012** | **MN818972** |
| *M. squarrosa* (Steph.) Heinrichs et al. | Brazil | Schäfer-Verwimp & Verwimp 14638 (JE) | KC313158 | KC313198 | KC313119 |
| *M. ulicina* (Taylor) Steph. | La Palma | Schäfer-Verwimp & Verwimp 24800 (GOET) | KC313154 | KC313194 | KC313115 |
| ***M. wallichiana* (Lehm.) R.L.Zhu & Y.M.Wei** | **India** | **Long 26477 (E)** | **MN822950** | **MN823013** | **MN818973** |

**Table S6** The nine defined areas of distribution of *Lejeunea* for the biogeographical analyses. A: the Neotropics; B: eastern North America; C: Europe; D: Africa (including Madagascar); E: continental Asia; F: tropical Asia; G: Australia-Pacific Islands; H: India; and I: western North America. Map was created through free and open access QGIS and modified in CorelDRAW by G.E.Lee.

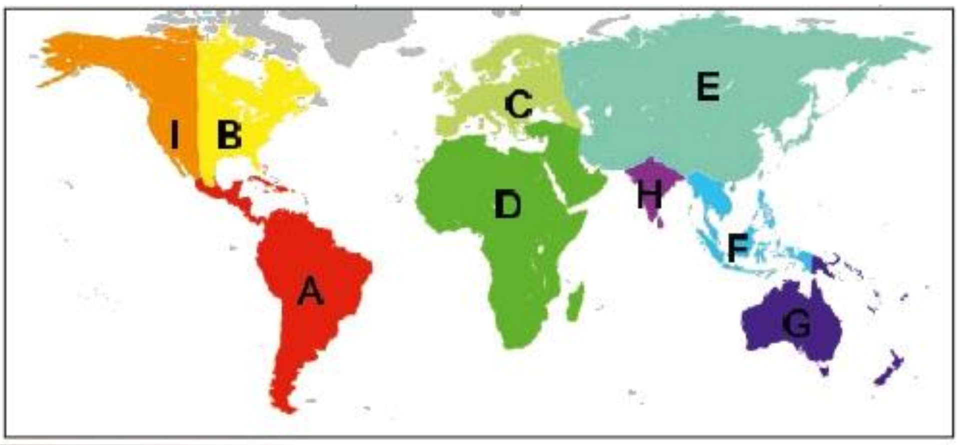


| Species | ABCDEFGHI (0: absent; 1: present) | References |
|---|---|---|
| *Lepidolejeunea cordifissa* | 100000000 | Piippo (1985) |
| *Lepidolejeunea cuspidata* | 100000000 | Piippo (1985) |
| *Lepidolejeunea eluta* | 100000000 | Piippo (1985), Gradstein et al. (2001) |
| *Lepidolejeunea involuta* | 100000000 | Piippo (1985), Gradstein et al. (2001) |
| *Lepidolejeunea delessertii* | 000100000 | Piippo (1985) |
| *Microlejeunea squarrosa* | 100000000 | Bischler et al. (1963); Schuster (1980) |
| *Microlejeunea wallichiana* | 000001010 | Mizutani (1964, 1971) |
| *Microlejeunea latitans* | 000000100 | Voucher specimen (New Zealand) |
| *Microlejeunea africana* | 000100000 | Voucher specimen (São Tomé and Príncipe), Wigginton (2004), Grolle (1995) |
| *Microlejeunea capillaris* | 100000000 | Bischler et al. (1963), Schuster (1980) |
| *Microlejeunea colombiana* | 100000000 | Bischler et al. (1963), Schuster (1980) |
| *Microlejeunea ulicina* | 011111110 | Mizutani (1971), Wang et al. (2011) |
| *Microlejeunea ankasica* | 000100000 | Voucher specimen (Príncipe Island), Wigginton (2004) |
| *Microlejeunea globosa* | 110100000 | Bischler et al. (1963), Schuster (1980) |
| *Microlejeunea punctiformis* | 000011110 | Mizutani (1961), Voucher specimen (Singapore), Wang et al. (2011), Long et al. (2014) |
| *Microlejeunea nyandaruensis* | 000100000 | Voucher specimen (Kenya) |
| *Microlejeunea kamerunensis* | 000100000 | Voucher specimen (Equatorial Guinea), Wigginton (2004) |
| *Microlejeunea bullata* | 110000000 | Bischler et al. (1963), Schuster (1980); Gradstein et al. (2001) |
| *Microlejeunea cystifera* | 100000000 | Bischler et al. (1963); Schuster (1980) |
| *Microlejeunea filicuspis* | 000101100 | Mizutani (1973) |
| *Microlejeunea fischeri* | 000100000 | Voucher specimen (Uganda) |
| *Harpalejeunea grandistipula* | 100000000 | Schuster (1999) |
| *Harpalejeunea uncinata* | 100000000 | Evans (1903), Bastos & Bôas-Bastos (2000) |
| *Harpalejeunea grandis* | 100000000 | Evans (1903); Gradstein et al. (2001) |
| *Harpalejeunea marginalis* | 100000000 | Voucher specimen (Chile) |
| *Harpalejeunea molleri* | 011000000 | Voucher specimen (Azores) |

| Species | ABCDEFGHI (0: absent; 1: present) | References |
|---|---|---|
| *Harpalejeunea stricta* | 110000000 | Evans (1903), Schuster (1967) |
| *Lejeunea acuta* | 000100000 | Voucher specimen (Kenya), Wigginton (2004) |
| *Lejeunea adpressa* | 110000000 | Gradstein & Costa (2003) |
| *Lejeunea lomana* | 000100000 | Voucher specimen (Réunion), Wigginton (2004) |
| *Lejeunea alata* | 000111100 | Lee (2013), Pócs & Wei (2017), Zhu & So (1999), Marline et al. (2012) |
| *Lejeunea amaniensis* | 000100000 | Voucher specimen (Kenya), Wigginton (2004), Grolle (1995), Marline et al. (2012) |
| *Lejeunea aquatica* | 000010000 | Mizutani (1961), Wang et al. (2011) |
| *Lejeunea asperrima* | 100000000 | Ilkiu-Borges (2005) |
| *Lejeunea asperula* | 000000100 | Mizutani (1976) |
| *Lejeunea bermudiana* | 110000000 | Reiner-Drehwald & Goda (2000) |
| *Lejeunea boryana* | 100000000 | Reiner-Drehwald & Goda (2000) |
| *Lejeunea cancellata* | 110000000 | Reiner-Drehwald (2000) |
| *Lejeunea capensis* | 100100000 | Giancotti & Vital (1989), Gradstein et al. (2001), Marline et al. (2012) |
| *Lejeunea catinulifera* | 100000000 | Gradstein & Costa (2003) |
| *Lejeunea cavifolia* | 011110010 | Mizutani (1971), Fariselli et al. (2018) |
| *Lejeunea cerina* | 100000000 | Reiner-Drehwald (1999, 2013) |
| *Lejeunea colensoana* | 000000100 | Renner et al. (2010) |
| *Lejeunea controversa* | 100000000 | Reiner-Drehwald & Goda (2000) |
| *Lejeunea curviloba* | 000010010 | Voucher specimen (Bhutan), So & Zhu (1998), Wang et al. (2011) |
| *Lejeunea debilis* | 100000000 | Gradstein & Costa (2003) |
| *Lejeunea deplanata* | 100000000 | Reiner-Drehwald (2010), Reiner-Drehwald & Grolle (2012) |
| *Lejeunea drehwaldii* | 100000000 | Heinrichs et al. (2012) |
| *Lejeunea eckloniana* | 001100000 | Voucher specimen (Madeira), Wigginton (2004) |
| *Lejeunea exilis* | 000111110 | Lee (2013), Pócs & Wei (2017), Zhu & Grolle (2003) |
| *Lejeunea flava* | 111111111 | Reiner-Drehwald (2000), Gradstein et al. (2001); Lee (2013), Pócs & Wei (2017), Wigginton (2004), Long et al. (2014), Marline et al. (2012) |
| *Lejeunea grossitexta* | 100000000 | Reiner-Drehwald & Goda (2000) |
| *Lejeunea helmsiana* | 000000100 | Renner et al. (2009) |
| *Lejeunea hibernica* | 001000000 | Voucher specimen (Ireland) |
| *Lejeunea holtii* | 001000000 | Voucher specimen (Madeira) |
| *Lejeunea intricata* | 100000000 | Gradstein & Costa (2003) |
| *Lejeunea isocalycina* | 100000000 | Gradstein & Costa (2003) |
| *Lejeunea japonica* | 000010000 | Mizutani (1961), Zhu & So (2001), Wang et al. (2011) |
| *Lejeuena laeta* | 100000000 | Reiner-Drehwald et al. (2018) |
| *Lejeunea laetevirens* | 111000000 | Reiner-Drehwald (2000) |
| *Lejeunea lamacerina* | 011000000 | Voucher specimen (Azores) |
| *Lejeunea compressiuscula* | 000001000 | Lee et al. (2018), Voucher specimen (Indonesia) |
| *Lejeunea microloba* | 000001100 | Lee (2013), Mizutani (1970) |
| *Lejeunea monimiae* | 100000000 | Reiner-Drehwald (2000) |
| *Lejeunea multidentata* | 100000000 | Voucher specimen (Dominican Republic) |
| *Lejeunea nepalensis* | 000000010 | Voucher specimen (Nepal) |
| *Lejeunea obtusangula* | 110000000 | Reiner-Drehwald (2000) |
| *Lejeunea oligoclada* | 100000000 | Reiner-Drehwald & Schäfer-Verwimp (2008a) |

| Species | ABCDEFGHI (0: absent; 1: present) | References |
|---|---|---|
| *Lejeunea oracola* | 000000100 | Renner et al. (2010) |
| *Lejeunea osculatiana* | 100000000 | Reiner-Drehwald & Schäfer-Verwimp (2008b) |
| *Lejeunea pallescens* | 100000000 | Reiner-Drehwald & Schäfer-Verwimp (2008b) |
| *Lejeunea parva* | 000011000 | Mizutani (1971), Lee et al. (2014), Wang et al. (2011), Shu et al. (2017) |
| *Lejeunea paucidentata* | 100000000 | Voucher specimen (Cuba) |
| *Lejeunea phyllobola* | 100100010 | Reiner-Drehwald (2000), Gradstein et al. (2001), Müller et al. (2011), Pócs (1995), Marline et al. (2012) |
| *Lejeunea pterigonia* | 100000000 | Gradstein & Costa (2003) |
| *Lejeunea puiggariana* | 100000000 | Reiner-Drehwald (2000) |
| *Lejeunea pulverulenta* | 100000000 | Gradstein & Costa (2003) |
| *Lejeunea ramosissima* | 000100100 | Voucher specimen (São Tomé and Príncipe), Wigginton (2004) |
| *Lejeunea ramulosa* | 100000000 | Reiner-Drehwald (2000) |
| *Lejeunea reflexistipula* | 100000000 | Gradstein & Costa (2003), Reiner-Drehwald (2005) |
| *Lejeunea rotundifolia* | 100000000 | Gradstein & Costa (2003) |
| *Lejeunea ruthii* | 010000000 | Voucher specimen (U.S.A.) |
| *Lejeunea sporadica* | 100000000 | Voucher specimen (Panama) |
| *Lejeunea subspathulata* | 100000000 | Ilkiu-Borges (2005) |
| *Lejeunea sulphurea* | 100000000 | Voucher specimen (Guadeloupe) |
| *Lejeunea tasmanica* | 000000100 | Voucher specimen (New Zealand) |
| *Lejeunea topoensis* | 100000000 | Gradstein & Reiner-Drehwald (2007) |
| *Lejeunea trinitensis* | 100100000 | Gradstein et al. (2001), Reiner-Drehwald & Grolle (2012) |
| *Lejeunea tumida* | 000000100 | Renner et al. (2010) |
| *Lejeunea umbilicata* | 000001100 | Lee (2013), Pócs & Wei (2017) |
| *Lejeunea cristulata* | 100000000 | Reiner-Drehwald (2000) |
| *Lejeunea neelgherriana* | 000010010 | Voucher specimen (Japan), Zhu & So (2000), Long et al. (2014) |
| *Lejeunea ptosimophylla* | 100000000 | Gradstein & Costa (2003), Reiner-Drehwald & Grolle (2012) |
| *Lejeunea conformis* | 000100000 | Voucher specimen (São Tomé) |
| *Lejeunea cyathophora* | 100000000 | Gradstein & Costa (2003) |
| *Lejeunea subplana* | 100000000 | Gradstein & Costa (2003) |
| *Lejeunea terricola* | 100000000 | Gradstein & Costa (2003) |
| *Lejeunea urbanii* | 100000000 | Gradstein & Costa (2003) |
| *Lejeunea coryantha* | 100000000 | Gradstein & Costa (2003) |
| *Lejeunea glaucescens* | 110000000 | Voucher specimen (Brazil) |
| *Lejeunea obtusata* | 000100000 | Voucher specimen (Kenya), Wigginton (2004), Marline et al. (2012) |
| *Lejeunea hepaticola* | 000100000 | Voucher specimen (Kenya) |
| *Lejeunea ibadana* | 000100000 | Voucher specimen (Príncipe Isl.), Wigginton (2004) |
| *Lejeunea dipterocarpa* | 000100000 | Voucher specimen (Equatorial Guinea), Wigginton (2004) |
| *Lejeunea eifrigii* | 000011010 | Lee (2013), Lee et al. (2011), Wang et al. (2011), Shu et al. (2017) |
| *Lejeunea albescens* | 000001100 | Lee (2013), Mizutani (1970) |
| *Lejeunea dipterota* | 000001000 | Lee (2013); Eifrig (1936) |

| Species | ABCDEFGHI (0: absent; 1: present) | References |
|---|---|---|
| *Lejeunea patriciae* | 000001100 | Lee (2013), Lee & Pócs (2018) |
| *Lejeunea mizutanii* | 000001100 | Lee (2013), Müller et al. (2016) |
| *Lejeunea lumbricoides* | 000001100 | Lee (2013), Pócs & Wei (2017) |
| *Lejeunea mimula* | 000001100 | Lee (2013), Pócs & Wei (2017) |
| *Lejeunea discreta* | 000011110 | Lee (2013), Pócs & Wei (2017), Singh et al. (2015), Zhu & So (1999), Long et al. (2014), Shu et al. (2017) |
| *Lejeunea cocoes* | 000011110 | Lee (2013), Pócs & Wei (2017), Mizutani (1963), Zhu & So (1999), Long et al. (2014), Shu et al. (2017) |
| *Lejeunea kinabalensis* | 000001100 | Lee (2013), Mizutani (1970) |
| *Lejeunea dimorpha* | 000001100 | Lee (2013), Lee & Pócs (2018), Shu et al. (2017) |
| *Lejeunea patersonii* | 000001100 | Lee (2013), Pócs & Wei (2017) |
| *Lejeunea pectinella* | 000001000 | Lee (2013), Mizutani (1970) |
| *Lejeunea pulchriflora* | 000101100 | Lee et al. (2016), Lee (2013), Pócs & Wei (2017) |
| *Lejeunea anisophylla* | 000111110 | Lee (2013), Pócs (2010), Pócs & Wei (2017), Müller et al. (2011), Wang et al. (2011), Long et al. (2014), Marline et al. (2012) |
| *Lejeunea tuberculosa* | 000111110 | Lee (2013), Pócs & Wei (2017), Zhu & So (1999), Müller et al. (2011), Wigginton (2004), Long et al. (2014), Shu et al. (2017), Grolle (1995), Marline et al. (2012) |
| *Lejeunea apiculata* | 000011110 | Lee (2013), Pócs & Wei (2017), Wang et al. (2011), Long et al. (2014) |
| *Lejeunea reinerae* | 000001100 | Ilkiu-Borges (2005), Pócs & Wei (2017) |
| *Lejeunea obscura* | 000011110 | Zhu & So (1999), Eifrig (1936), Wang et al. (2011), Long et al. (2014) |
| *Lejeunea globosiflora* | 100000000 | Gradstein & Costa (2003), Gradstein & Cuvertino (2015) |
| *Lejeunea kuerscheriana* | 000100000 | Voucher specimen (Kenya) |
| *Lejeunea caracensis* | 100000000 | Gradstein & Costa (2003) |
| *Lejeunea stephaniana* | 000001000 | Lee (2013), Mizutani (1964) |
| *Lejeunea leratii* | 000011100 | Lee (2013), Wang et al. (2011) |
| *Lejeunea papilionacea* | 000111110 | Lee (2013), Pócs & Wei (2017), Mizutani (1972), Müller et al. (2011), Wigginton (2004), Long et al. (2014), Zhu & Grolle (2001), Marline et al. (2012) |
| *Lejeunea micholitzii* | 000001100 | Lee (2013), Pócs & Wei (2017) |
| *Lejeunea brenanii* | 000100000 | Voucher specimen (São Tomé), Wigginton (2004) |
| *Lejeunea sordida* | 000011110 | Lee (2013), Pócs & Wei (2017), Lee & Pócs (2018), Wang et al. (2011) |
| *Lejeunea wightii* | 000011010 | Lee (2013), Mizutani (1964), Zhu & So (1999), Wang et al. (2011), Long et al. (2014) |
| *Lejeunea fleischeri* | 000001000 | Lee (2013), Mizutani (1972) |
| *Lejeunea utriculata* | 000001000 | Lee (2013), Mizutani (1970) |
| *Lejeunea thallophora* | 000001000 | Eifrig (1936), Voucher specimen (Indonesia) |
| *Lejeunea gradsteinii* | 000001000 | Lee (2013), Lee et al. (2011) |
| *Lejeunea compacta* | 000011000 | Lee (2013), Mizutani (1961), Zhu & So (1999), Wang et al. (2011) |
| *Lejeunea stenodentata* | 000001100 | Voucher specimen (Papua New Guinea) |
| *Lejeunea subolivacea* | 000000010 | Mizutani (1964, 1965), Srivastava & Agarwal (1987) |
| *Lejeunea stevensiana* | 000010010 | Voucher specimen (China), Zhu & So (1999) |

| Species | ABCDEFGHI (0: absent; 1: present) | References |
|---|---|---|
| *Lejeunea konosensis* | 000010000 | Voucher specimen (China) |
| *Lejeunea alata* var. *patriciae* | 000100100 | Voucher specimen (Samoa), Marline et al. (2012) |
| *Lejeunea soae* | 000010010 | Voucher specimen (China) |
| *Lejeunea sikorae* | 000100000 | Voucher specimen (Madagascar), Grolle (1995) |
| *Lejeunea tapajosensis* | 100000000 | Reiner-Drehwald (2000); Reiner-Drehwald & Grolle (2012) |
| *Lejeunea isophylla* | 000100000 | Voucher specimen (Madagascar), Grolle (1995), Marline et al. (2012) |

**Table S7** The time-stratified biogeographic model that specified constraints on area connections used for DEC analyses.

Time slice 1

| | A | B | C | D | E | F | G | H | I |
|---|---|---|---|---|---|---|---|---|---|
| A | 1 | 1 | 0 | 0 | 0 | 0 | 0 | 0 | 0 |
| B | 1 | 1 | 0 | 0 | 0 | 0 | 0 | 0 | 1 |
| C | 0 | 1 | 1 | 0 | 1 | 1 | 0 | 0 | 0 |
| D | 0 | 0 | 0 | 1 | 1 | 0 | 0 | 0 | 0 |
| E | 0 | 0 | 1 | 1 | 1 | 0 | 0 | 1 | 1 |
| F | 0 | 0 | 1 | 0 | 1 | 1 | 0 | 0 | 0 |
| G | 0 | 0 | 0 | 0 | 0 | 1 | 1 | 0 | 0 |
| H | 0 | 0 | 0 | 0 | 1 | 0 | 1 | 1 | 0 |
| I | 0 | 1 | 0 | 0 | 1 | 0 | 0 | 0 | 1 |

Time slice 2

| | A | B | C | D | E | F | G | H | I |
|---|---|---|---|---|---|---|---|---|---|
| A | 1 | 1 | 0 | 0 | 0 | 0 | 0 | 0 | 0 |
| B | 1 | 1 | 0 | 0 | 0 | 0 | 0 | 0 | 1 |
| C | 0 | 1 | 1 | 0 | 1 | 1 | 0 | 0 | 0 |
| D | 0 | 0 | 0 | 1 | 1 | 0 | 0 | 0 | 0 |
| E | 0 | 0 | 1 | 1 | 1 | 0 | 0 | 1 | 1 |
| F | 0 | 0 | 1 | 0 | 1 | 1 | 0 | 0 | 0 |
| G | 0 | 0 | 0 | 0 | 0 | 1 | 1 | 0 | 0 |
| H | 0 | 0 | 0 | 0 | 1 | 0 | 1 | 1 | 0 |
| I | 0 | 1 | 0 | 0 | 1 | 0 | 0 | 0 | 1 |

Time slice 3

| | A | B | C | D | E | F | G | H | I |
|---|---|---|---|---|---|---|---|---|---|
| A | 1 | 1 | 0 | 0 | 0 | 0 | 0 | 0 | 0 |
| B | 1 | 1 | 0 | 0 | 0 | 0 | 0 | 0 | 1 |
| C | 0 | 1 | 1 | 0 | 1 | 1 | 0 | 0 | 0 |
| D | 0 | 0 | 0 | 1 | 1 | 0 | 0 | 0 | 0 |
| E | 0 | 0 | 1 | 1 | 1 | 0 | 0 | 1 | 1 |
| F | 0 | 0 | 1 | 0 | 1 | 1 | 0 | 0 | 0 |
| G | 0 | 0 | 0 | 0 | 0 | 1 | 1 | 0 | 0 |
| H | 0 | 0 | 0 | 0 | 1 | 0 | 1 | 1 | 0 |
| I | 0 | 1 | 0 | 0 | 1 | 0 | 0 | 0 | 1 |

Time slice 4

| | A | B | C | D | E | F | G | H | I |
|---|---|---|---|---|---|---|---|---|---|
| A | 1 | 1 | 0 | 0 | 0 | 0 | 0 | 0 | 0 |
| B | 1 | 1 | 0 | 0 | 0 | 0 | 0 | 0 | 1 |
| C | 0 | 1 | 1 | 0 | 1 | 1 | 0 | 0 | 0 |
| D | 0 | 0 | 0 | 1 | 1 | 0 | 0 | 0 | 0 |

| | | | | | | | | | |
|---|---|---|---|---|---|---|---|---|---|
| E | 0 | 0 | 1 | 1 | 1 | 0 | 0 | 1 | 1 |
| F | 0 | 0 | 1 | 0 | 1 | 1 | 0 | 0 | 0 |
| G | 0 | 0 | 0 | 0 | 0 | 1 | 1 | 0 | 0 |
| H | 0 | 0 | 0 | 0 | 1 | 0 | 1 | 1 | 0 |
| I | 0 | 1 | 0 | 0 | 1 | 0 | 0 | 0 | 1 |

Time slice 5

| | A | B | C | D | E | F | G | H | I |
|---|---|---|---|---|---|---|---|---|---|
| A | 1 | 1 | 0 | 0 | 0 | 0 | 0 | 0 | 0 |
| B | 1 | 1 | 0 | 0 | 0 | 0 | 0 | 0 | 1 |
| C | 0 | 1 | 1 | 0 | 1 | 1 | 0 | 0 | 0 |
| D | 0 | 0 | 0 | 1 | 1 | 0 | 0 | 0 | 0 |
| E | 0 | 0 | 1 | 1 | 1 | 0 | 0 | 1 | 1 |
| F | 0 | 0 | 1 | 0 | 1 | 1 | 0 | 0 | 0 |
| G | 0 | 0 | 0 | 0 | 0 | 1 | 1 | 0 | 0 |
| H | 0 | 0 | 0 | 0 | 1 | 0 | 1 | 1 | 0 |
| I | 0 | 1 | 0 | 0 | 1 | 0 | 0 | 0 | 1 |

Time slice 6

| | A | B | C | D | E | F | G | H | I |
|---|---|---|---|---|---|---|---|---|---|
| A | 1 | 1 | 0 | 0 | 0 | 0 | 0 | 0 | 0 |
| B | 1 | 1 | 0 | 0 | 0 | 0 | 0 | 0 | 1 |
| C | 0 | 1 | 1 | 0 | 1 | 1 | 0 | 0 | 0 |
| D | 0 | 0 | 0 | 1 | 1 | 0 | 0 | 0 | 0 |
| E | 0 | 0 | 1 | 1 | 1 | 0 | 0 | 1 | 1 |
| F | 0 | 0 | 1 | 0 | 1 | 1 | 0 | 0 | 0 |
| G | 0 | 0 | 0 | 0 | 0 | 1 | 1 | 0 | 0 |
| H | 0 | 0 | 0 | 0 | 1 | 0 | 1 | 1 | 0 |
| I | 0 | 1 | 0 | 0 | 1 | 0 | 0 | 0 | 1 |

**Figure S1** Maximum Likelihood (ML) phylogeny of *Lejeunea* based on three markers from nuclear and plastid DNA. ML bootstrap probabilities ≥ 50 are shown at branches; a star indicates a Posterior Probability ≥ 0.95.

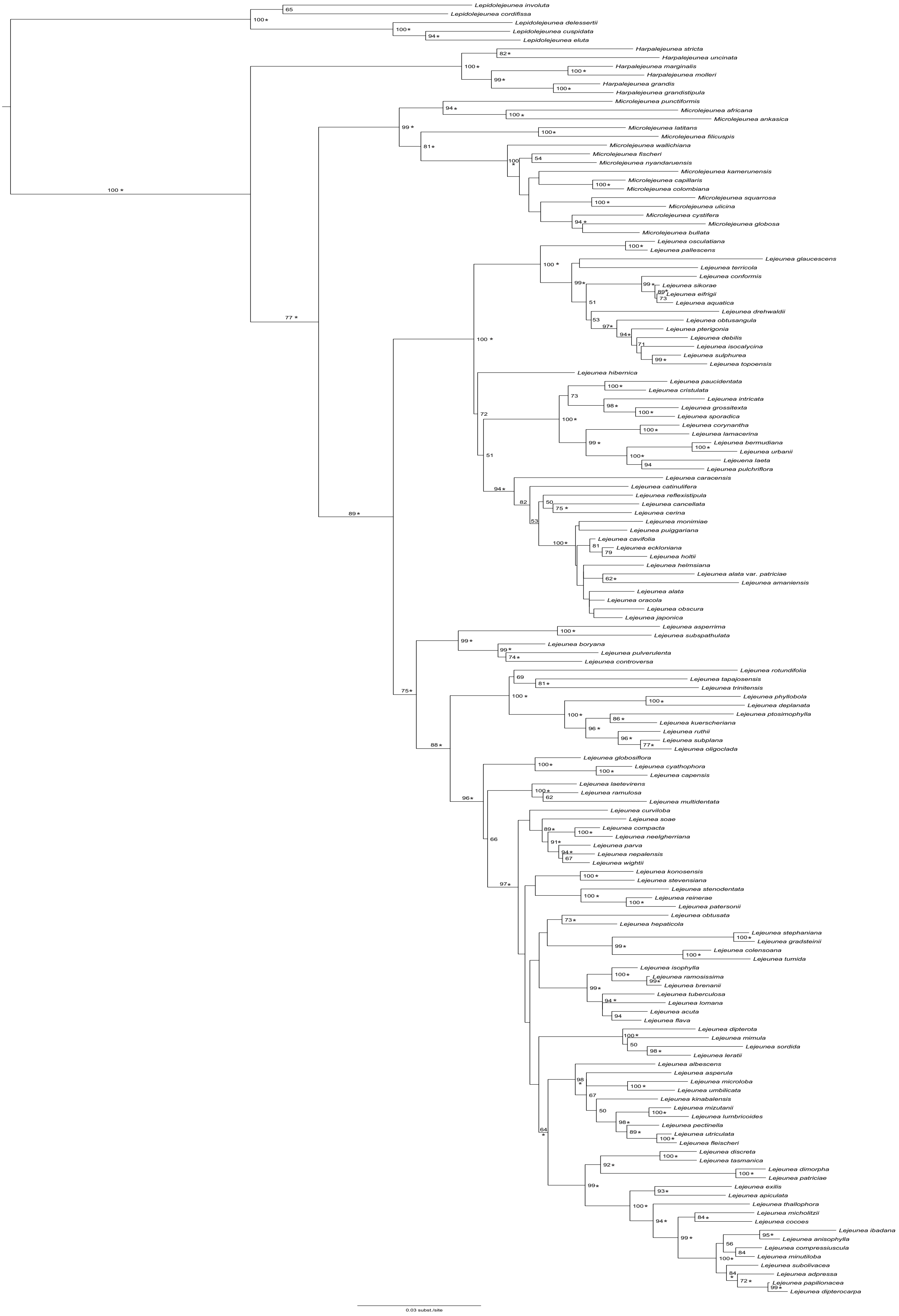

**Figure S2** Reconstruction of the biogeographical history for *Lejeunea* using an unconstrained DEC model as inferred from BioGeoBEARS.

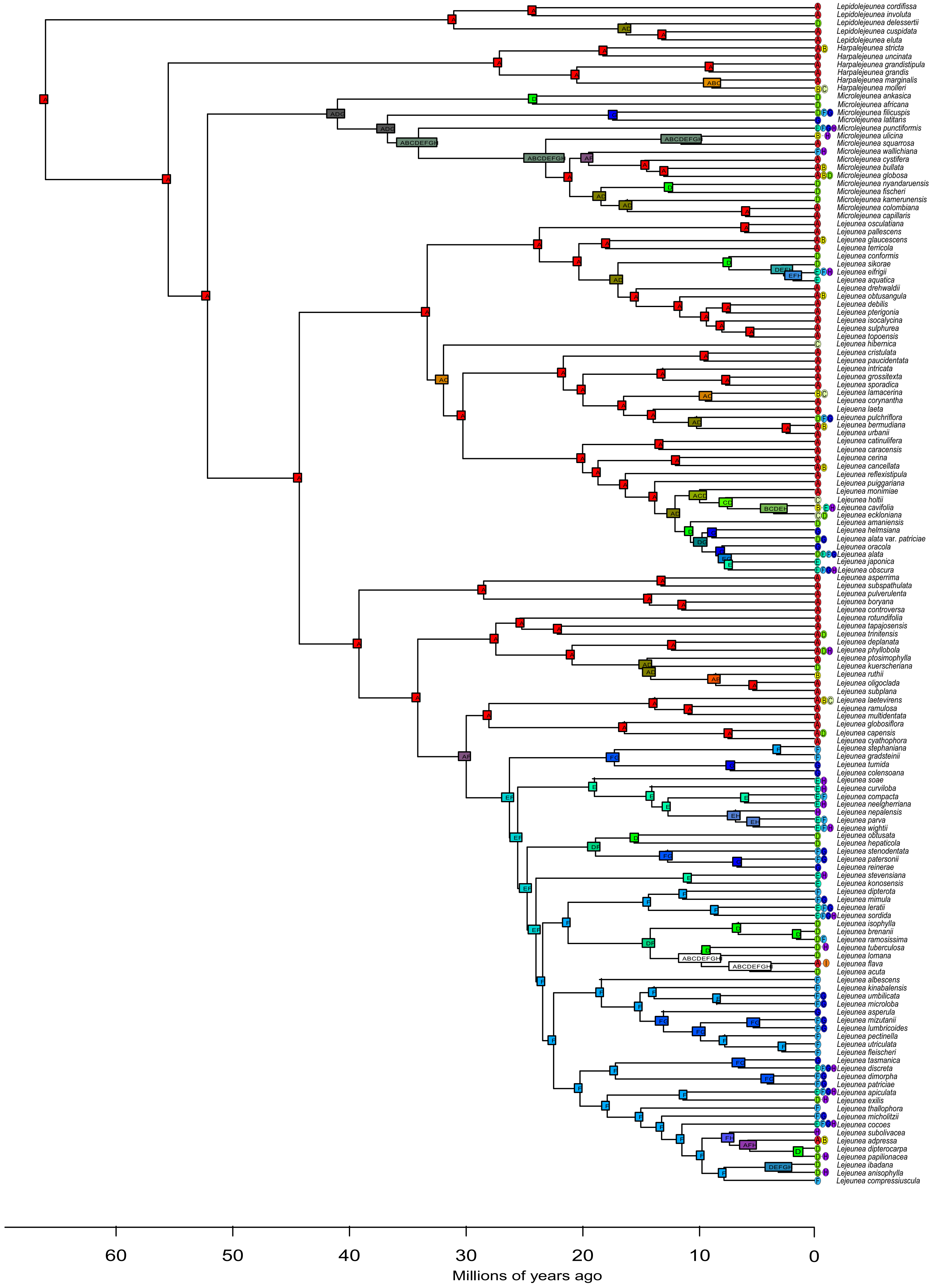

**Figure S3** The pie chart from the reconstruction of the biogeographical history for *Lejeunea* using a time-stratified DEC model as inferred from BioGeoBEARS.

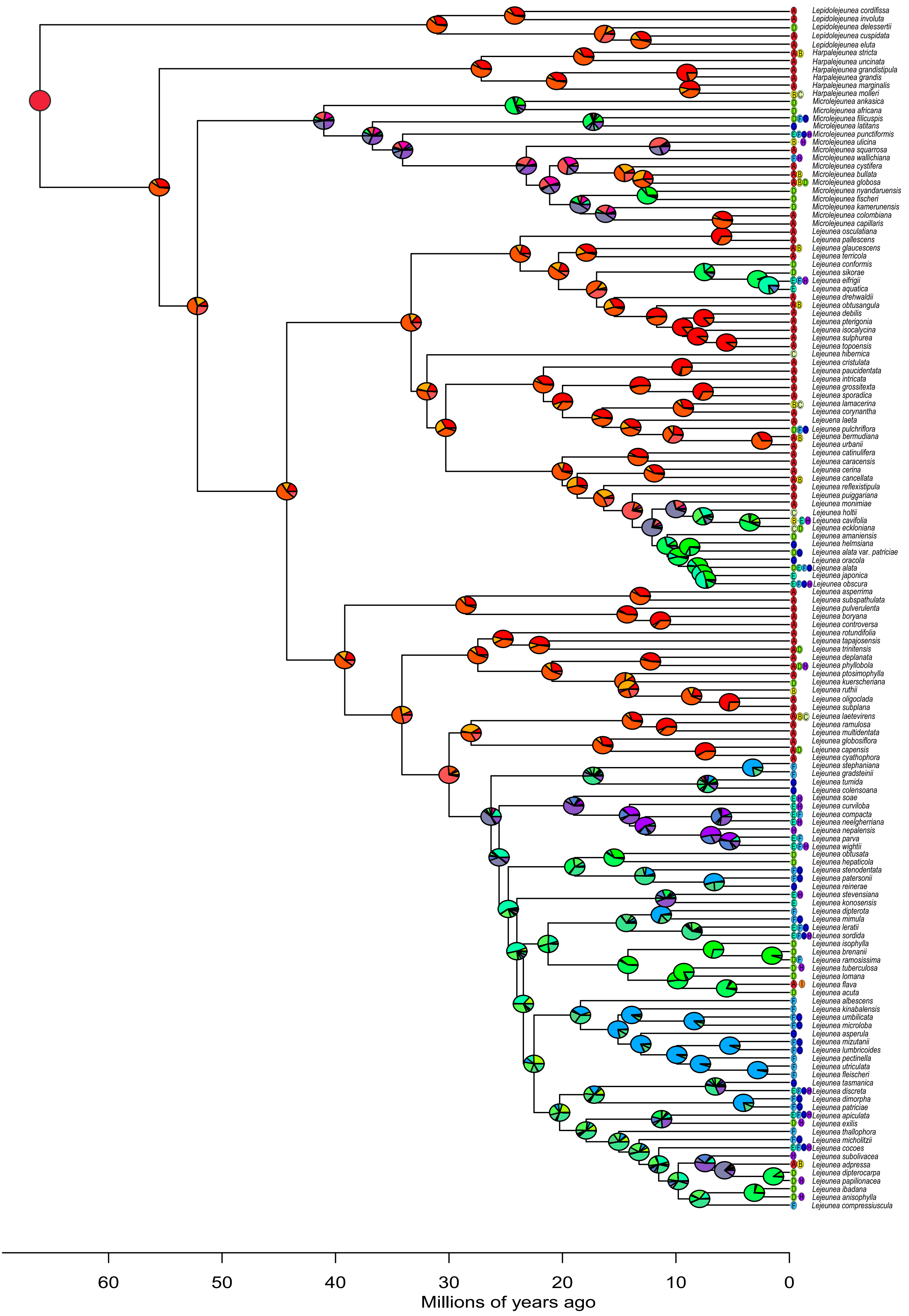

**Figure S4** Robustness of BiSSE models with simulation test. For the simulation, the difference in fit (ΔAIC) between the best BiSSE model and the null model is shown with the red vertical line for real data, and in gray for simulated datasets.

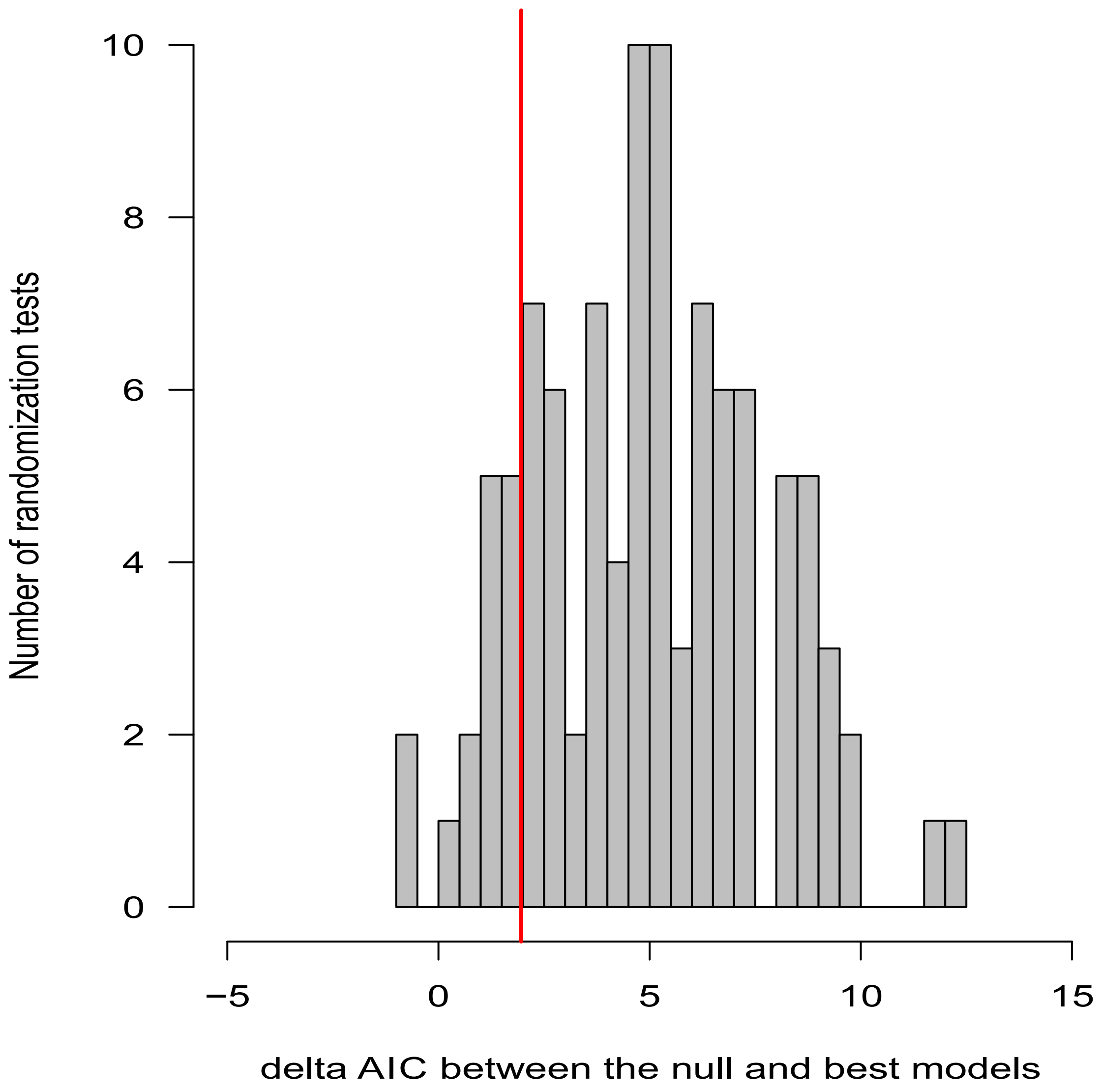

**Figure S5** Reconstruction of the biogeographical history for *Lejeunea* as inferred from BioGeoBEARS, DIVALIKE model.

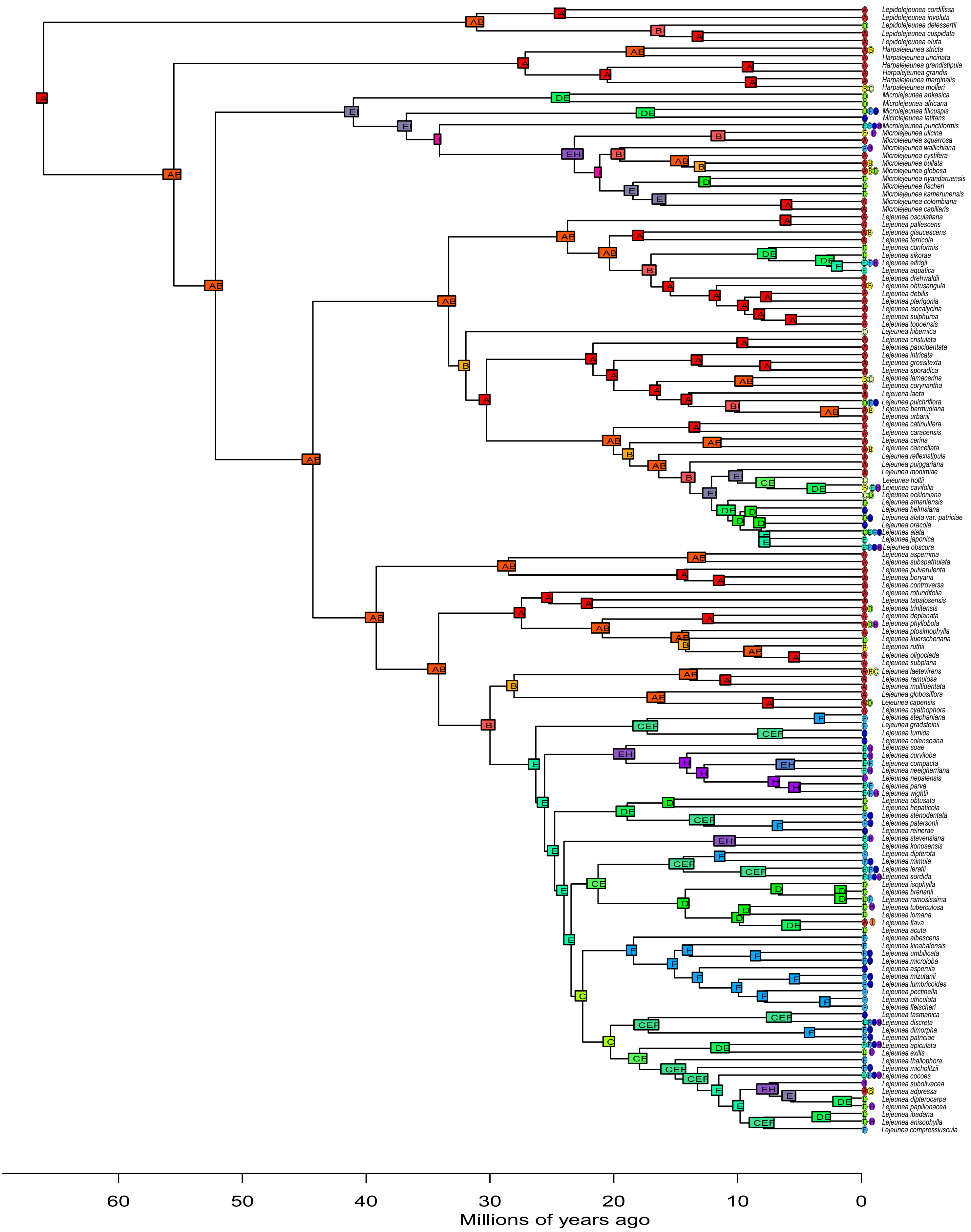

Supplement: Supplementary file 1 — Supplementary Information [file 41598_2020_71039_MOESM1_ESM.pdf]
